# Cross-Reactivity, Epitope Mapping, and Potency of Monoclonal Antibodies to Class 5 Fimbrial Tip Adhesins of Enterotoxigenic Escherichia coli

**DOI:** 10.1128/IAI.00246-20

**Published:** 2020-10-19

**Authors:** Yang Liu, Sakina Shahabudin, Sami Farid, Lanfong H. Lee, Annette L. McVeigh, Milton Maciel, Steven T. Poole, Matthew Broadman, Michael G. Prouty, Stephen J. Savarino

**Affiliations:** a Henry M. Jackson Foundation for the Advancement of Military Medicine, Bethesda, Maryland, USA; b Enteric Diseases Department, Naval Medical Research Center, Silver Spring, Maryland, USA; c U.S. Naval Medical Research Unit No. 3, Cairo, Egypt; d Department of Pediatrics, Uniformed Services University of the Health Sciences, Bethesda, Maryland, USA; University of California San Diego School of Medicine

**Keywords:** ETEC, enterotoxigenic *Escherichia coli*, fimbrial tip adhesin, monoclonal antibodies

## Abstract

Enterotoxigenic Escherichia coli (ETEC) is a leading diarrheagenic bacterial pathogen among travelers and children in resource-limited regions. Adherence to host intestinal cells mediated by ETEC fimbriae is believed to be a critical first step in ETEC pathogenesis. These fimbriae are categorized into related classes based on sequence similarity, with members of the class 5 fimbrial family being the best characterized. The eight related members of the ETEC class 5 fimbrial family are subdivided into three subclasses (5a, 5b, and 5c) that share similar structural arrangements, including a fimbrial tip adhesin. However, sequence variability among the class 5 adhesins may hinder the generation of cross-protective antibodies. To better understand functional epitopes of the class 5 adhesins and their ability to induce intraclass antibody responses, we produced 28 antiadhesin monoclonal antibodies (MAbs) to representative adhesins CfaE, CsbD, and CotD, respectively. We determined the MAb cross-reactivities, localized the epitopes, and measured functional activities as potency in inhibition of hemagglutination induced by class 5 fimbria-bearing ETEC. The MAbs’ reactivities to a panel of class 5 adhesins in enzyme-linked immunosorbent assays (ELISAs) revealed several reactivity patterns, including individual adhesin specificity, intrasubclass specificity, intersubclass specificity, and class-wide cross-reactivity, suggesting that some conserved epitopes, including two conserved arginines, are shared by the class 5 adhesins. However, the cross-reactive MAbs had functional activities limited to strains expressing colonization factor antigen I (CFA/I), coli surface antigen 17 (CS17), or CS1, suggesting that the breadth of functional activities of the MAbs was more restricted than the repertoire of cross-reactivities measured by ELISA. The results imply that multivalent adhesin-based ETEC vaccines or prophylactics need more than one active component to reach broad protection.

## Abstract (precis)

Enterotoxigenic Escherichia coli (ETEC) is a leading diarrheagenic bacterial pathogen among travelers and children in resource-limited regions. Adherence to host intestinal cells mediated by ETEC fimbriae is believed to be a critical first step in ETEC pathogenesis. These fimbriae are categorized into related classes based on sequence similarity, with members of the class 5 fimbrial family being the best characterized. The eight related members of the ETEC class 5 fimbrial family are subdivided into three subclasses (5a, 5b, and 5c) that share similar structural arrangements, including a fimbrial tip adhesin.

## INTRODUCTION

Enterotoxigenic Escherichia coli (ETEC) is a major cause of watery diarrhea among travelers and young children in low to middle income countries (1–3). The adherence of ETEC to host intestinal cells via colonization factors (CFs) and the subsequent secretion of enterotoxins are the major initial steps in its pathogenesis, and thus, much of the current efforts to develop an ETEC vaccine have focused mainly on these virulence factors (4). While the development of a vaccine against human ETEC has been complicated by the serological diversity of more than 25 known CFs (5), many of these fimbriae are closely related, based on their sequence similarities (6). The ETEC class 5 fimbrial family consists of eight members divided into three subclasses, 5a (colonization factor antigen I [CFA/I], coli surface antigen 4 [CS4], and CS14), 5b (CS1, CS17, and CS19), and 5c (CS2) (5, 7), some of which are highly prevalent in human-pathogenic isolates (8).

In the past 2 decades, studies on these class 5 fimbriae have revealed their molecular assembly and functional components. Specifically, each class 5 fimbria is composed of more than 1,000 pilus major subunits and one or two tip-localized minor subunits (9–11), which are noncovalently connected through a donor strand complementation mechanism utilized by many other Gram-negative bacterial pili (10, 12, 13). Our group and others have demonstrated that the minor subunits of the class 5 fimbriae are essential components for the bacterial adherence, functioning as fimbrial tip adhesins. This is supported by findings that a single point mutation, a change of R to A at position 181 (R181A), in CooD (CS1 adhesin) and CfaE (CFA/I adhesin) abolished homologous bacterial binding to erythrocytes and intestinal cells (14–16) and that rabbit antibodies to CfaE reduced the binding of CFA/I-expressing (CFA/I^+^) ETEC to Caco-2 cells and inhibited hemagglutination induced by CFA/I^+^ ETEC (7, 15). Furthermore, the antibodies to the N-terminal half of CfaE were more effective in blocking the CFA/I^+^ ETEC binding to the host cells than were the antibodies to the C-terminal half of the adhesin (7). In addition, human monoclonal antibodies (MAbs) to the putative receptor binding site of CfaE not only fully inhibited hemagglutination, diminished ETEC adhesion to Caco-2 cells, and reduced homologous ETEC colonization in the adult mouse model (17) but also demonstrated efficacy in a nonhuman primate model when challenged with the H10407 strain (18). Importantly, we have demonstrated that antibodies against CfaE are protective against CFA/I^+^ ETEC challenge in the infant suckling mouse model (19) and the nonhuman primate (*Aotus nancymaae*) model (20). Moreover, human subjects administered oral bovine immunoglobulin from cows immunized with CfaE or CFA/I were protected from CFA/I^+^ ETEC challenge (21, 22). Taken together, these findings support the development of an ETEC vaccine based on the fimbrial tip adhesins.

However, a major challenge to the development of a broadly protective adhesin-based ETEC vaccine is the antigenic and sequence variability among the class 5 adhesins. We have previously showed that anti-CfaE and anti-CsbD (CS17 adhesin) rabbit antisera could inhibit hemagglutination induced by ETEC expressing heterologous CFs within each respective subclass (7, 15). However, the sequence identities among the eight class 5 adhesins are much lower than those among adhesins within subclasses 5a and 5b (7). To determine if cross-reactive functional epitopes are present among the class 5 adhesins, we generated 28 monoclonal antibodies from mice immunized with each of three representative class 5 adhesins, CfaE, CsbD, and CotD (CS2 adhesin), and studied the cross-reactivities, epitopes, and potencies of these MAbs.

## RESULTS

### Cross-reactive patterns of MAbs to heterologous class 5 fimbrial tip adhesins.

**FIG 1:**
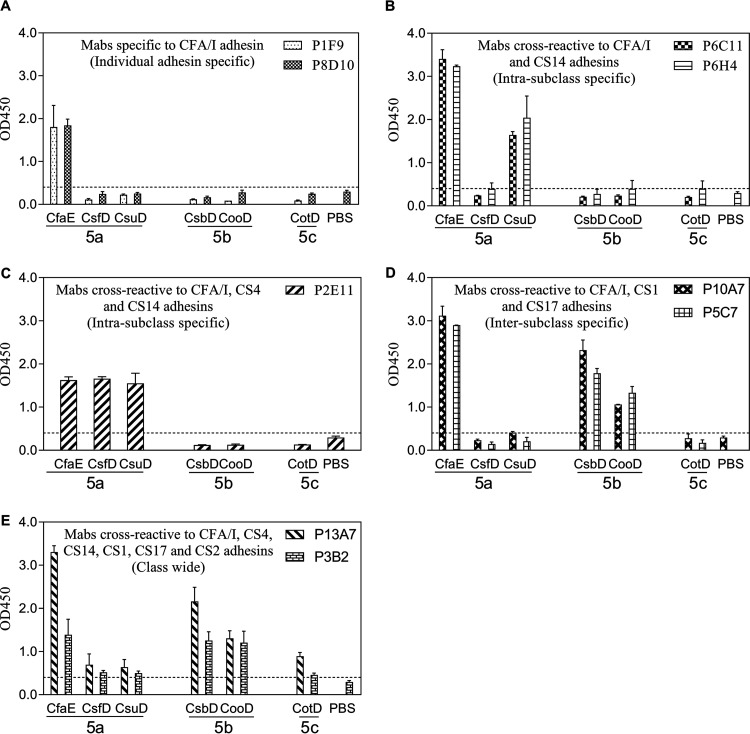
Anti-CfaE MAb cross-reactivities to a panel of class 5 adhesins in the ELISAs. MAbs with similar cross-reactivity patterns are grouped together. (A) MAbs specific to the CFA/I adhesin. (B) MAbs cross-reactive to the CFA/I and CS14 adhesins. (C) MAb cross-reactive to the CFA/I, CS4, and CS14 adhesins. (D) MAbs cross-reactive to the CFA/I, CS1, and CS17 adhesins. (E) MAbs cross-reactive to the CFA/I, CS4, CS14, CS1, CS17, and CS2 adhesins. The bars and error bars represent the mean OD values and standard deviations from at least two repeated assays. The dashed lines represent the limit of detection in the anti-CfaE MAb ELISAs, which was the sum of the average PBS background level and three times the standard deviation.

We examined the reactivity pattern of each MAb by enzyme-linked immunosorbent assay (ELISA), aiming to identify broadly reactive MAbs. The raw optical density (OD) measurements in the ELISAs are displayed in Fig. 1 and 3, and we defined positive reactivity as mean OD values of adhesins that were greater than the sum of the mean OD value and three times the standard deviation for phosphate-buffered saline (PBS). The binding of MAbs to homologous and heterologous class 5 adhesins is summarized in Table 1. The 28 antiadhesin MAbs exhibited several cross-reactive patterns. Specifically, we observed individual-adhesin-specific, intrasubclass-specific, intersubclass-specific, and class-wide cross-reactivities. Two anti-CfaE MAbs, P1F9 and P8D10, reacted only to the immunogen CfaE (Fig. 1A, individual adhesin specific), while P6C11 and P6H4 were cross-reactive to a second class 5a adhesin, CsuD (Fig. 1B, intrasubclass specific) and P2E11 showed similar reactivities to all three class 5a adhesins (CFA/I, CS4, and CS14) (Fig. 1C, intrasubclass specific). Moreover, anti-CfaE MAbs P5C7 and P10A7 were cross-reactive to two class 5b adhesins, CsbD (CS17) and CooD (CS1) (Fig. 1D, intersubclass specific). Notably, two anti-CfaE MAbs, P13A7 and P3B2, reacted to all tested class 5 adhesins with various intensities (Fig. 1E, class-wide reactivity).

**TABLE 1:** Summary of antiadhesin MAb reactivities to class 5 adhesins in ELISAs

MAb	Reactivity to indicated adhesin of subclass^*a*^:					
	5a			5b		5c
	CfaE	CsfD	CsuD	CsbD	CooD	CotD
Anti-CfaE MAbs						
P8D10	+	−	−	−	−	−
P6C11	+	−	+	−	−	−
P6H4	+	−	+	−	−	−
P10A7	+	−	−	+	+	−
P5C7	+	−	−	+	+	−
P2E11	+	+	+	−	−	−
P3B2	+	+	+	+	+	+
P13A7	+	+	+	+	+	+
P1F9	+	−	−	−	−	−

Anti-CsbD MAbs						
P7C2	−	−	−	+	+	−
P9A5	−	−	−	+	+	−
P2H6	−	−	−	+	+	−
P6G1	−	−	−	+	+	−
P2A9	−	−	−	+	+	−
P1F7	−	−	−	+	+	−
P9E11	+	+	+	+	+	−
P7F9	+	+	+	+	+	+
P5A12	−	−	−	+	+	−
P9D12	−	−	−	+	+	−
P7F12	−	−	−	+	+	−

Anti-CotD MAbs						
P7F6	−	−	−	−	−	+
P3F4	−	−	−	−	−	+
P6B8	−	−	−	−	−	+
P3D11	−	−	−	−	−	+
P9A10	+	+	+	−	−	+
P9G7	−	+	+	−	−	+
P2B8	−	−	−	−	−	+
P12A2	+	−	+	−	−	+

a +, positive reactivity; −, negative reactivity.

**FIG 2:**
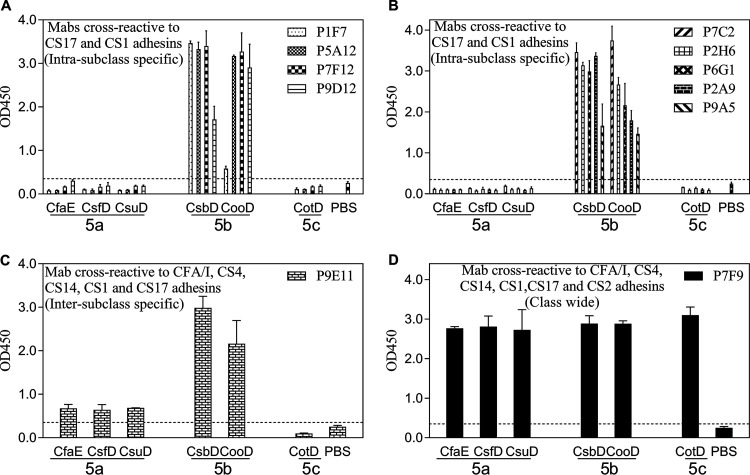
Anti-CsbD MAb cross-reactivities to a panel of class 5 adhesins in the ELISAs. MAbs with similar cross-reactivity patterns are grouped together. (A and B) MAbs cross-reactive to the CS17 and CS1 adhesins. (C) MAb cross-reactive to the CFA/I, CS4, CS14, CS17, and CS1 adhesins. (D) MAb cross-reactive to the CFA/I, CS4, CS14, CS1, CS17, and CS2 adhesins. The bars and error bars represent the mean OD values and standard deviations from at least two repeated assays. The dashed lines represent the limit of detection in the anti-CsbD MAb ELISAs, which was the sum of the average PBS background level and three times the standard deviation.

All 11 anti-CsbD MAbs were cross-reactive to the CS1 adhesin CooD, presumably due to the high sequence identity (97%) between CsbD and CooD (Fig. 2). Anti-CsbD MAb P9E11 was also reactive to all three class 5a adhesins (Fig. 2C). Surprisingly, the other anti-CsbD MAb, P7F9, was broadly reactive to all class 5 adhesins examined (Fig. 2D).

**FIG 3:**
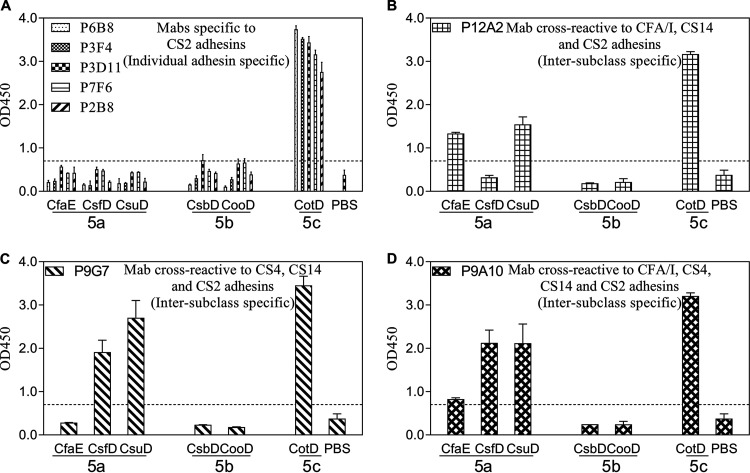
Anti-CotD MAb cross-reactivities to a panel of class 5 adhesins in the ELISAs. MAbs with similar cross-reactivity patterns are grouped together. (A) MAbs specific to the CS2 adhesin. (B) MAb cross-reactive to the CFA/I, CS14, and CS2 adhesins. (C) MAb cross-reactive to the CS4, CS14, and CS2 adhesins. (D) MAb cross-reactive to the CFA/I, CS4, CS14, and CS2 adhesins. The bars and error bars represent the mean OD values and standard deviations from at least two repeated assays. The dashed lines represented the limit of detection in the anti-CotD MAb ELISAs, which was the sum of the average PBS background level and three times the standard deviation.

Five of eight anti-CotD MAbs reacted only to the immunogen CotD (Fig. 3A). Among the other three cross-reactive anti-CotD MAbs, P12A2 was cross-reactive to two class 5a adhesins, CfaE and CsuD (Fig. 3B), whereas P9G7 had additional reactivities to CsfD and CsuD (Fig. 3C). The third anti-CotD MAb, P9A10, was reactive to all class 5a adhesins (Fig. 3D).

### MAb epitope mapping, epitope features, domain specificities, and isotypes.

To further understand the distinctive reactivity patterns and cross-reactive epitopes of the 28 antiadhesin MAbs to the class 5 adhesins, we next performed epitope mapping on the MAbs with an initial focus on those with cross-reactivity to the heterologous adhesins. To map the binding epitopes of each MAb, we used a combination of ELISAs (Fig. S1 to S4 in the supplemental material) and functional hemagglutination inhibition (HAI) assays (see below). The results are summarized in Table 2. In the ELISAs, the identity of a residue within the binding epitope was inferred by a detectable difference in reactivities to the MAb between the native adhesin and the adhesin’s allelic variants or mutants. In the HAI assays, an epitope-specific residue was inferred by an obvious difference in inhibitory concentrations of the MAbs to multiple allelic variants. Specifically, since CfaE/R67A and CfaE/R181A demonstrated reduced binding to anti-CfaE MAbs P8D10, P6C11, P6H4, P10A7, and P5C7 (Fig. S1A to E), we inferred that the binding epitopes of these five anti-CfaE MAbs included residues R67 and R181 in CfaE, which are known to be involved in host cell binding (14, 16, 23) (Table 2). Two anti-CfaE MAbs, P10A7 and P5C7, showed moderate hemagglutination inhibition activity against the CS17-expressing (CS17^+^) strain WS6788A (Table 3), but the same inhibition was not observed against the CS17^+^ strain WS4240A (harboring the CsbD L85I allelic variation) (Table S1), suggesting that S86 in CfaE, sequence-aligned with L85 in CsbD (Fig. S5), is a potential residue in the epitopes of anti-CfaE MAbs P10A7 and P5C7 (Table 2).

**TABLE 2:** Isotypes, epitope features, domain specificities, and epitope residues of antiadhesin MAbs

MAb	Isotype	Epitope feature(s)	Domain specificity^*a*^	Epitope residue(s)
Anti-CfaE MAbs				
P8D10	IgG1	Conformational	AD	R67, R181
P6C11	IgG2b	Conformational	AD	R67, R181
P6H4	IgG1	Conformational	AD	R67, R181
P10A7	IgG1	Conformational	AD	R67, S86^*b*^, R181
P5C7	IgG1	Conformational	AD	R67, S86^*b*^, R181
P2E11	IgG1	Conformational	PD	ND^*c*^
P3B2	IgG1	Conformational	PD	ND
P13A7	IgG1	Linear	PD	ND
P1F9	IgG1	Conformational	PD	ND
Anti-CsbD MAbs				
P7C2	IgG1	Conformational	AD	ND
P9A5	ND	Conformational	AD	ND
P2H6	IgG1	Conformational	AD	T84
P6G1	IgG1	Conformational	AD	ND
P2A9	IgG1	Conformational	AD	ND
P1F7	IgG1	Conformational	AD	H144
P9E11	ND	Conformational	AD	ND
P7F9	IgG1	Linear/conformational	AD	S88, R181, Y182
P5A12	IgG1	Conformational	AD	ND
P9D12	ND	Linear	PD	ND
P7F12	IgG1	Linear	PD	ND
Anti-CotD MAbs				
P7F6	IgG1	Conformational	AD	ND
P3F4	IgG2a	Conformational	AD	ND
P6B8	IgG1	Conformational	AD	R69, R184
P3D11	IgG1	Conformational	AD	ND
P9A10	IgG1	Linear	AD	ND
P9G7	IgG1	Linear	PD	ND
P2B8	IgG1	Linear	PD	ND
P12A2	IgG1	Linear	PD	ND

a AD, adhesin domain; PD, pilin domain.

b The epitope residue was identified by hemagglutination inhibition assay.

c ND, not determined by the methods used in this study.

**TABLE 3:** Functional activities of antiadhesin MAbs measured by the minimum concentrations to inhibit hemagglutination induced by eight ETEC strains expressing class 5 fimbriae

MAb	MIC (μg/ml) against MRHA caused by ETEC strain (in parentheses) expressing indicated fimbriae of subclass^*a*^:							
	5a			5b				5c
	CFA/I (H10407)	CS4 (BANG10-SP)	CS14 (WS3294A)	CS17 (WS6788A)	CS17 (LSN02-013966/a)^*b*^	CS17 (WS4240A)^*c*^	CS1 (WS1974A)	CS2 (C91f)
Anti-CfaE MAbs								
P8D10^*d*^	1.2	—	—	—	—	ND	—	—
P6C11^*d*^	1.2	—	—	—	—	ND	—	—
P6H4^*d*^	1.6	—	—	—	—	ND	—	—
P10A7^*d*^	0.8	—	—	8.0	187.5	—	—	—
P5C7^*d*^	1.2	—	—	16.0	—	—	—	—
P2E11	31	—	—	—	—	ND	—	—
P3B2	3	—	—	12.0	31.3	ND	188	—
P13A7	>400	>400	>400	13.0	>400	ND	>400	>400
P1F9	1.5	—	—	—	—	ND	—	—
Anti-CsbD MAbs								
P7C2^*d*^	—	—	—	1.0	1.0	2.0	4	—
P9A5^*d*^	—	—	—	4.0	3.9	ND	4	—
P2H6^*d*^	—	—	—	4.0	31.3	375	31	—
P6G1^*d*^	—	—	—	8.0	15.6	ND	16	—
P2A9^*d*^	—	—	—	8.0	15.6	ND	16	—
P1F7^*d*^	—	—	—	0.3	—	ND	—	—
P9E11^*d*^	—	—	—	4.0	—	ND	—	—
P7F9^*d*^	—	—	—	—	—	ND	—	—
P5A12^*d*^	—	—	—	—	—	—	—	—
P9D12	—	—	—	63	—	ND	—	—
P7F12	—	—	—	—	—	ND	—	—
Anti-CotD MAbs								
P7F6^*d*^	—	—	—	—	—	ND	—	0.5
P3F4^*d*^	—	—	—	—	250	ND	—	1.0
P6B8^*d*^	—	—	—	—	—	ND	—	3.0
P3D11^*d*^	—	—	—	—	—	ND	—	16.0
P9A10^*d*^	—	—	—	—	—	ND	—	188
P9G7	—	—	—	—	—	ND	—	—
P2B8	—	—	—	—	250	ND	—	250
P12A2	—	—	—	—	—	ND	—	125

a MAbs with MICs of ≤10 μg/ml, 10 to 100 μg/ml, and 100 to 250 μg/ml were defined in this study as having strong, moderate, and low functional activity, respectively. —, the MIC was greater than 250 μg/ml; ND, not determined (the experiments were not performed).

b The CS17^+^ LSN02-013966/A strain contains the CsbD/L85I/H144A allelic variation from the sequence of the CS17^+^ WS6788A strain.

c The CS17^+^ WS4240A strain contains the CsbD/L85I allelic variation from the sequence of the CS17^+^ WS6788A strain.

d The MAb was adhesin domain specific.

Of the anti-CsbD MAbs, P2H6 reacted less to CsbD from strain E20738A (harboring N62S/S74T/T84N/L85R/H144A/Y145N/Y293H allelic variations) or CsbD/T84N/L85R than to the reference CsbD, CsbD adhesin domain (AD), CsbD from strain LSN02-013966/A (harboring L85I/H144A allelic variations), or CsbD/H144A/Y145N (Fig. S2C), and thus, it is likely that P2H6 binds an epitope involving T84 in CsbD (Table 2). A second anti-CsbD MAb, P1F7, bound nominally to CsbD/H144A/Y145N or CsbD/L85I/H144A compared to its binding to the reference CsbD or CsbD/T84N/L85R (Fig. S2F), and thus, we reasoned that the P1F7 binding epitope contained the H144 residue (Table 2). Interestingly, another anti-CsbD MAb, P7F9, bound much less to CfaE/T91V, CfaE/R181A, and CfaE/R182A than to the reference CfaE (Fig. S2H); hence, the P7F9 epitope included S88, R181, and Y182 (Table 2), because T91, R181, and R182 in CfaE are aligned in sequence with S88, R181, and Y182 in CsbD, respectively.

**FIG 4:**
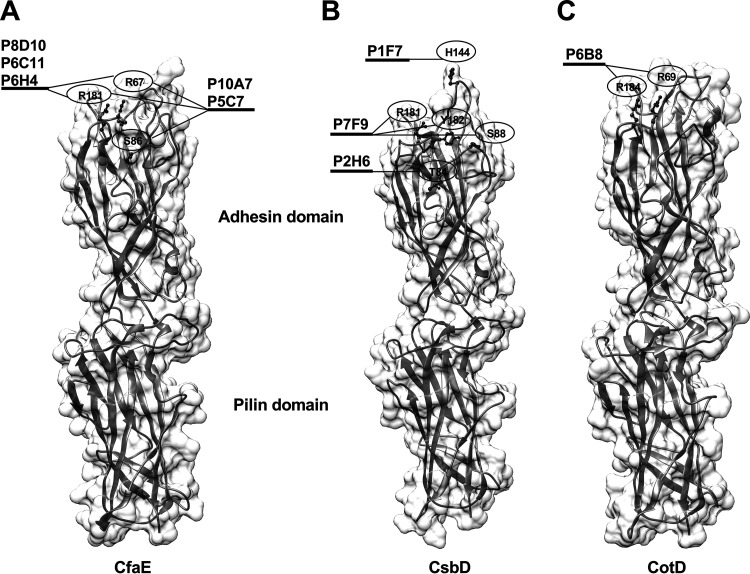
Spatial locations of residues recognized by nine antiadhesin MAbs. (A) Residues (ball-and-stick view) within epitopes of anti-CfaE MAbs P8D10, P6C11, P6H4, P10A7, and P5C7 were mapped onto the CfaE crystal structure (ribbon and surface view; PDB code 2HB0). The receptor binding site of CfaE was located in the adhesin domain and included residues R67 and R181. (B and C) Residues within epitopes of anti-CsbD MAbs P2H6, P1F7, and P7F9 (B) and anti-CotD MAb P6B8 (C) were mapped onto the CsbD and CotD structure models, respectively.

In the anti-CotD MAb ELISA performed with specific CotD mutants (Fig. S4), the anti-CotD MAb P6B8 had nominal reactivity to CotD/R69A and CotD/R184A, so the epitope of P6B8 included R69 and R184 residues (Table 2). The identified epitope residues were mapped onto the CfaE crystal structure (Fig. 4A) and the respective structural models of CsbD and CotD (Fig. 4B and C), which were *in silico* generated using MODELLER (24), with PDB code 2HB0 as a template. The structural models of CsbD and CotD resembled the CfaE structure, with adhesin and pilin domains stacking together. Due to the limited number of adhesin allelic variants and mutants, we were only able to infer one or more epitope residues for nine MAbs. All epitope residues identified in this study were located within or near the putative receptor binding domains of the adhesins.

The epitope features (conformational or linear), domain specificities, and isotypes of the MAbs were determined to complement the epitope analysis, and the results are listed in Table 2. Compared to their binding to the native adhesins (immunogens) in the ELISAs (Fig. S1 to S3), 20 MAbs showed reduced binding to the heat-denatured adhesins, suggesting that these recognize conformational epitopes (Table 2). Based on the reactivity of each MAb to the respective adhesin domains and adhesin domain mutants (Fig. S1 to S3), 19 of the 28 MAbs were adhesin domain specific (Table 2). Specifically, 5 of 9 anti-CfaE MAbs, 9 of 11 anti-CsbD MAbs, and 5 of 8 anti-CotD MAbs were specific to the adhesin domains of CfaE, CsbD, and CotD, respectively. In addition, we confirmed that the four anti-CfaE MAbs that were not reactive to the CfaE adhesin domain showed reactivity to the CfaE pilin domain in the ELISA (Fig. S6). Among the 28 MAbs, 23 were determined to be isotype IgG1 by IsoQuick strips (Sigma) (Table 2), consistent with mouse IgG isotype distribution (25).

### HAI activity of antiadhesin MAbs.

The presence of cross-reactive MAbs to heterologous class 5 adhesins prompted us to investigate whether the cross-reactivity patterns observed in the ELISAs would be retained in the functional hemagglutination inhibition (HAI) assay. To test the potency and functional cross-reactivity of each MAb, we determined the minimum concentrations of MAbs needed to inhibit mannose-resistant hemagglutination (MRHA) of bovine erythrocytes elicited by eight ETEC strains expressing class 5 CFs (Table 3). All five anti-CfaE adhesin domain-specific MAbs (P8D10, P6C11, P6H4, P10A7, and P5C7) were very potent inhibitors of hemagglutination induced by a CF-homologous ETEC strain (CFA/I^+^ strain H10407), with the MICs being less than 10 μg/ml. Among them, P10A7 and P5C7 also showed strong or moderate functional cross-reactivity to the heterologous CS17^+^ ETEC strain WS6788A, suggesting that the three residues (R67, S86, and R181) recognized by P10A7 and P5C7 were functional epitope residues shared between CfaE and CsbD. Of the four anti-CfaE pilin domain-specific MAbs, P3B2, P1F9, and P2E11 had high or moderate hemagglutination inhibition (HAI) activities against the CF-homologous strain, which was unexpected since the putative receptor-binding domain of CfaE has been localized to the adhesin domain of CfaE (Fig. 4) (23). Interestingly, P3B2 even exhibited moderate and low inhibitory effects on the heterologous CS17^+^ and CS1-expressing (CS1^+^) strains, respectively. Surprisingly, one anti-CfaE pilin domain-specific MAb, P13A7, did not show homologous HAI activity up to 400 μg/ml; however, it displayed moderate heterologous HAI to the CS17*^+^* strain.

Among the anti-CsbD MAbs, seven of nine MAbs specific to the adhesin domain were highly potent inhibitors of hemagglutination induced by the CF-homologous strain (CS17^+^ WS6788A). These MAbs were generally less potent inhibitors for the other two CS17^+^ strains, presumably based on the allelic variation (Table S1). Five CsbD MAbs, P7C2, P9A5, P2H6, P6G1, and P2A9, also showed functional cross-reactivity to the CS1-heterologous class 5b CF-expressing strain (Table 3), suggesting that there are common functional epitopes, such as T84 in CsbD, shared between CsbD and CooD. No HAI activity was observed for the other two adhesin domain-specific MAbs (P7F9 and P5A12) to any of the eight ETEC strains within the normal range of tested concentrations (≤250 μg/ml), which was unexpected because P7F9 was shown to be broadly reactive to all six adhesins by ELISA (Table 1), and its epitope included the conserved R181 (Table 2). Two MAbs (P9D12 and P7F12) specific to the CsbD pilin domain had moderate or low potency in inhibiting MRHA induced by the CF-homologous strain, and neither of them exhibited any HAI activity against the CF-heterologous strain.

Among six anti-CotD MAbs specific to the adhesin domain, three (P7F6, P3F4, and P6B8) were highly potent inhibitors of MRHA induced by the CF-homologous ETEC strain; however, the other three MAbs (P3D11, P9A10, and P9G7) showed moderate or low levels of HAI against the CF-homologous ETEC strain. Two anti-CotD MAbs (P2B8 and P12A2) specific to the pilin domain displayed low HAI activities against the CF-homologous strain. None of the anti-CotD MAbs exhibited significant functional cross-reactivity to the CF-heterologous class 5 CF-expressing ETEC strains.

## DISCUSSION

The eight class 5 colonization factors have been shown to be expressed in approximately 30% of clinically isolated ETEC strains (8), and thus, coverage of class 5 CFs is critical for a multivalent ETEC vaccine. We have previously shown that orally delivered antibodies against the prototype class 5 fimbrial tip adhesin, CfaE, were protective against homologous ETEC challenge in human volunteers (21). However, it is unclear whether one class 5 adhesin would confer heterologous protection across the entire class or within subclasses. In the present study, we produced and characterized 28 MAbs recognizing a representative adhesin from each of the three class 5 subclasses. The MAb cross-reactivity patterns determined by ELISA showed individual-adhesin-specific, intrasubclass-specific, intersubclass-specific, and class-wide cross-reactivities. About two-thirds of the MAbs had epitopes residing in the adhesin domain, among which nine MAb epitopes were localized to the upper pole of the class 5 adhesins and included two conserved arginines. Functional activities measured in the HAI assay indicated that the adhesin domain-specific MAbs in general had higher homologous HAI activities than the pilin domain-specific MAbs. Heterologous HAI activities were observed in a few MAbs with limited coverage. Furthermore, the breadth of the functional cross-reactivities of the MAbs measured by the HAI assay were more restricted than the repertoire of cross-reactivities shown by the ELISA.

Among 17 MAbs displaying strong homologous HAI activity, 15 were specific to the adhesin domains, which was consistent with the finding in our previous study that the rabbit IgG Fab antibodies to the N-terminal half of CfaE were more potent in inhibiting CFA/I^+^ ETEC hemagglutination and binding to Caco-2 cells than those to the C-terminal half of the adhesin (7). These results are in agreement with other data implicating the adhesin domains as harboring the host cell receptor binding sites (14, 16, 23). The receptor binding sites of class 5 adhesins were first identified with alanine mutations of a few positively charged residues in CooD and CfaE, the results of which suggested that R181 in the adhesins was essential for hemagglutination induced by CS1^+^ and CFA/I^+^ ETEC, respectively (14). Furthermore, a study using an *in vitro* human intestine culture model indicated that R181 in CfaE played a critical role in the colonization of CFA/I^+^ ETEC (16). Based on the structure of CfaE, we identified a cluster of conserved positively charged residues around R181 in CfaE, including R67, H140, and R182, that were responsible for agglutination of human erythrocytes and, thus, defined this region as the receptor binding pocket (10). In the current study, all five adhesin domain-specific anti-CfaE MAbs with epitopes that included the R67 and R181 residues had strong homologous HAI activities, confirming that these two residues in CfaE are essential for hemagglutination. Two adhesin domain-specific anti-CsbD MAbs, P2H6 and P1F7, with epitopes in the vicinity of R67 and R181 showed strong homologous HAI responses, suggesting that the upper pole region of CsbD served as the receptor binding site and that this may be a universal feature for all class 5 adhesins. However, one exception was the anti-CsbD P7F9 MAb, whose epitope mapped to the upper pole, including R181, but which had low homologous HAI activity. One explanation is that the mouse spleen cell used to generate P7F9 hybridomas had not been through the affinity maturation (26) and the affinity of P7F9 was too low to elicit any HAI activity, as other studies have shown that the affinity of MAbs to certain antigens is positively correlated with their functionality (27, 28).

Functional antibodies against either receptor binding pockets of antigens or remote conserved regions can have distinct mechanisms of action. Antibodies against the receptor binding pocket in the hemagglutinin of influenza virus disrupt host-pathogen interaction, resulting in hemagglutination inhibition, though these antibodies are typically strain specific (29). Interestingly, antibodies directed at the conserved stem regions of hemagglutinin homotrimers, located far from the receptor binding pocket, were also neutralizing, though more broadly, with mechanisms thought to involve blocking the hemagglutinin conformational changes associated with virus-host cell membrane fusion (29). In our study, in addition to the 13 of 19 adhesin domain-specific MAbs that exhibited strong homologous HAI activities, we also identified two anti-CfaE pilin domain-specific MAbs that exhibited unexpectedly high homologous HAI activities. We previously showed that increased shear stress could activate CfaE into a high-affinity binding state (30), and partial disruption of the interface between the adhesin and pilin domains of CfaE leads to activation and a significant structural shift in the pilin domain (31). The MAbs specific to the pilin domain of CfaE could bind and lock the native conformation of the pilin domain, prevent structural changes under the shear stress generated by the rocking in the HAI assay, and hold CfaE in the low-affinity binding state, resulting in hemagglutination inhibition. In particular, the anti-CfaE pilin domain-specific MAb P3B2 showed HAI activity with not only the homologous CFA/I^+^ strain, but also heterologous CS17^+^ and CS1^+^ strains. This MAb could have epitopes that include residues in the donor strand, which is in the pilin domain and conserved across class 5 fimbriae (23). This hypothesis was supported by results showing that the P3B2 MAb was reactive to a peptide within the donor strand in the peptide ELISA (data not shown) and results from the previous study suggesting that a monoclonal antibody against the N-terminal 25 residues of CFA/I subunits, which serve as the donor strand in the pilin domain, had HAI activity to CFA/I^+^, CS1^+^, or CS4^+^ ETEC and blocked those ETEC from binding to the Caco-2 cells (32).

Among the 28 MAbs generated in this study, 21 cross-reacted to at least one of the heterologous class 5 adhesins in ELISA; however, functional cross-reactivity was only observed in 9 MAbs. Since some MAbs may cross-react with epitopes not involved in hemagglutination, it was expected that only a subset would show heterologous HAI activity. We demonstrated in the current study that among 11 anti-CsbD MAbs that were all cross-reactive to the CS1 adhesin CooD in ELISA, only 5 anti-CsbD MAbs had both homologous HAI activity and heterologous HAI (CS1) activity. Notably, among MAbs with both cross-reactivity in ELISA and homologous HAI activity, the breadth of the heterologous HAI activities of the MAbs was more limited than the repertoire of cross-reactivities shown by the ELISA. One obvious example was anti-CfaE MAb P3B2, which was cross-reactive to other five class 5 adhesins tested but showed heterologous HAI activities only to CS17^+^ and CS1^+^ ETEC. The results suggested that the hemagglutination inhibition assay was more discriminating than ELISA in distinguishing subtle differences of epitopes in the class 5 adhesins recognized by the MAbs.

Although the results from the current study imply a lack of broad functional epitopes within class 5 adhesins from ETEC, it is possible for an ETEC vaccine to achieve broad coverage through various approaches. Formulations consisting of multiple ETEC antigens (33) or sequences (34) have elicited broad functional responses in polyclonal antisera. Mice primed and boosted with different ETEC colonization factors generated high IgG titers to both immunogens (35). Chimeric malaria antigens harnessing polymorphisms within the inhibitory epitopes produced functional inhibition against both related and unrelated strains (36), suggesting that a similar engineering approach can be applied to class 5 adhesins from ETEC. Nevertheless, evaluating the broad functional serum polyclonal antibody response is an indirect assessment of the contribution or role of individual monoclonal antibodies in such sera.

Recent studies demonstrated that human anti-CfaE MAbs with epitopes localized to the putative receptor binding pocket showed strong inhibition of CFA/I^+^ ETEC binding in cell-based assays using erythrocytes and Caco-2 cells, reduced homologous bacterial colonization in the adult mouse model (17), and prevented diarrhea induced by the H10407 strain in the nonhuman primate model (18). The anti-adhesin MAbs identified in the present study could be humanized, developed, and tested as immunoprophylactic products to increase coverage of other predominant ETEC strains. Further, the MAbs described here can be used to develop assays identifying immunodominant epitopes of class 5 adhesins with competitive ELISA and potent functional epitopes of class 5 adhesins with competitive HAI assay using specific MAbs and sera from human volunteers immunized with CfaE in clinical trials (clinicaltrials.gov registration numbers NCT01644565 and NCT01922856). Nevertheless, our results with a limited number of MAbs suggested that a multivalent ETEC prophylactic or vaccine may require more than one active component due to a lack of cross-reactive functional epitopes in the class 5 adhesins.

## MATERIALS AND METHODS

### Construction, expression, and purification of adhesin proteins.

The genes encoding donor strand-complemented proteins dscCsuD, dscCsfD, dscCsbD, dscCooD, and dscCotD (CS14, CS4, CS17, CS1, and CS2 adhesins, respectively) were cloned into pET24(a)+ (Novagen) between the XhoI and NdeI sites, similarly to the construction of the dscCfaE plasmid (15). The C-terminal donor strand of each adhesin was from the N-terminal 15 to 19 residues of the CsuA, CsfA, CsbA, CooA, and CotA (CS14, CS4, CS17, CS1, and CS2 pilins, respectively) mature sequences. dscCfaE and dscCsbD served as templates for site-directed mutagenesis (QuikChange; Stratagene) to introduce point mutation(s) at the specific residues. Each of these recombinant adhesin and adhesin mutant plasmids, which include an in-frame C-terminal hexahistidine tag, was transformed into E. coli strain BL21(DE3) for expression. Cell growth, induction, harvest, lysis, and protein purification were similar to the procedures previously reported for dscCfaE (15). Briefly, the cells were grown in APS super broth (Difco) at 32°C with kanamycin and induced with 1 mM IPTG (isopropyl β-d-1-thiogalactopyranoside). Cells were harvested and disrupted using a microfluidizer. After centrifugation of the cell lysate, the soluble proteins were purified by nickel affinity and cation exchange chromatography sequentially. The purified proteins were pooled and concentrated. The purity and concentration of the antigens were determined by densitometry and bicinchoninic acid (BCA) assay (Pierce), respectively. The antigens used in the ELISAs are listed in Table S1 in the supplemental material.

### Mouse hybridoma generation.

Female BALB/c mice were immunized with 4 doses (5 μg per dose) of each adhesin (dscCfaE, dscCsbD, or dscCotD) at 2-week intervals. Three days after the last immunization, splenocytes of the immunized mice were fused at 1:10 with mouse myeloma cell line P3NS1 in the presence of polyethylene glycol. After the fused cells were incubated with HAT selective medium (Gibco) for 10 days in tissue culture microtiter plates, the supernatants of stable hybridomas were tested for antibody production on ELISA plates coated with each adhesin. The culture supernatants were diluted at a 1:1 ratio with phosphate-buffered saline, pH 7.4, plus 0.05% Tween 20 and 0.1% bovine serum albumin (PBST-BSA) and added into plates. Bound antibodies were detected by incubation with goat anti-mouse IgG horseradish peroxidase conjugates diluted at 1:1,500 in PBST-BSA, and the optical densities (OD) at 450 nm were measured after incubation with the *ortho*-phenylenediamine and peroxide substrate. The OD values of the positive hybridomas were at least 0.1 higher than the background levels. There were 28 positive hybridomas. Nine of them were anti-dscCfaE, 11 were anti-dscCsbD, and 8 were anti-dscCotD.

### MAb purification.

About 40-ml amounts of supernatant of the hybridoma cell cultures were adjusted to pH 8.0 with sodium hydroxide and applied to 0.5 ml of protein G resin (Genscript) at 0.5 ml/min. After washing with 15 ml of PBS, pH 7.4, the MAbs were eluted with 5 ml of 100 mM glycine, pH 2.5. The eluate was immediately neutralized by 1 M Tris, pH 8.5. The fractions containing purified MAbs were dialyzed against water, lyophilized, and resuspended in PBS, pH 7.4. The final MAb concentrations were determined by BCA assay (Pierce). Each purified monoclonal antibody was examined on reducing and denaturing 15% SDS-PAGE gels. Each protein had bands corresponding to immunoglobulin heavy chain and light chain. All monoclonal antibodies had purity of over 90% based on the densitometry analysis of the lane.

### ELISA.

Enzyme-linked immunosorbent assay (ELISA) procedures were performed as previously described (31). Briefly, antigens were diluted in PBS, pH 7.4, and coated on a 96-well microtiter plate with 100 μl of each adhesin and mutant at 2 μg/ml. Each condition was repeated at least two times. After the plate was incubated at 37°C for 1 h, each well was washed three times with 250 μl of PBS. Then, each well was blocked with 250 μl of PBS with 5% fetal calf serum at 37°C for 1 h. After washing three times with 250 μl of PBS, 0.05% Tween 20 (PBST), 100 μl of each mouse MAb at 2 μg/ml was added to each well and the plate incubated at 37°C for 1 h. The plate was washed five times with 250 μl of PBST, 100 μl of goat anti-mouse IgG horseradish peroxidase-conjugated secondary antibodies was added to each well, and the plate incubated at 37°C for 2 h. After washing three times with 250 μl of PBST, 100 μl of *ortho*-phenylenediamine substrate was added to each well and the plate incubated at 25°C for 20 min. Optical densities at 450 nm were measured by using a plate reader. The mean OD values, standard deviations, and limit of detection (the sum of the mean OD and 3 times the standard deviation from PBS) were plotted on the graphs. The limit of detection was used to discern the MAb reactivity differences between adhesins and PBS and between wild-type adhesins and mutants. The complete ELISA data are available in Fig. S1, S2, S3, S4, and S6, while only selected ELISA data are presented in Results.

### HAIs.

The hemagglutination inhibition (HAI) assays were performed as previously described (7). The wild-type class 5 fimbria-bearing ETEC strains used in this study were H10407 (CFA/I), WS1974A (CS1), C91f (CS2), BANG10-SP (CS4), WS3294A (CS14), LSN02-013966/A (CS17), WS6788A (CS17), and WS4240A (CS17). Briefly, bacteria were resuspended in PBS with 0.5% d-mannose (PBSM) until the OD at 650 nm reached 40. The minimal hemagglutination titer (MHT) was determined by mixing 25 μl of each serial 2-fold bacterial dilution with equal volumes (25 μl of each) of 3% washed bovine erythrocytes and PBSM in the ceramic tile wells. The tile was rocked on ice for 20 min. The second-highest bacterial dilution (one titer higher than the MHT) showing positive mannose-resistant hemagglutination (MRHA) was used as the bacterial working solution. To determine the HAI activity or the MIC of each MAb, a serial 2-fold antibody dilution was made from a starting concentration of 500 μg/ml and incubated with an equal volume (25 μl of each) of the bacterial working solution at room temperature for 20 min. Twenty-five microliters of 3% washed bovine erythrocytes was then added into the wells, and the tile was rocked on ice for 20 min. The hemagglutination was visually inspected and scored as follows: negative, no MRHA activity; 1+, weak reaction; 2+, moderate reaction; 3+, strong reaction; 4+, instantaneous reaction involving all erythrocytes. The MIC was expressed as the lowest concentration of the MAb which completely inhibited MRHA (i.e., no MRHA activity). The MICs reported in Table 3 are the average values from at least two repeated assays.

## Supplementary Material

Supplemental file 1
