# Supplementary material for: Cross-Reactivity, Epitope Mapping, and Potency of Monoclonal Antibodies to Class 5 Fimbrial Tip Adhesins of Enterotoxigenic Escherichia coli
Source: Infect Immun. 2020 Oct 19;88(11):e00246-20. doi: 10.1128/IAI.00246-20 (PMC7573445; doi:10.1128/IAI.00246-20)

Supplemental Table

Table S1. Table of antigens used in the ELISA.

Protein antigens	Sequence origin	Note
Class 5a antigens		
CfaE	CFA/I (H10407)	dsc_{19}CfaE (reference)
CfaE AD	CFA/I (H10407)	Adhesin domain (residues 23-202) of CfaE
CfaE PD	CFA/I (H10407)	Pilin domain (residues 203-383) of CfaE
CfaE/R67A	CFA/I (H10407)	CfaE mutant R67A
CfaE/T91I	CFA/I (10F2)	CfaE has an allelic variation of T91I in the CFA/I allelic strain 10F2
CfaE/A128V	CFA/I (WS4437A-1)	CfaE has an allelic variation of A128V in the CFA/I allelic strain WS4437A-1
CfaE/Q142R	CFA/I (SMJ344)	CfaE has an allelic variation of Q142R in the CFA/I allelic strain SMJ344
CfaE/R181A	CFA/I (H10407)	CfaE mutant R181A
CfaE/R182A	CFA/I (H10407)	CfaE mutant R182A
CsfD	CS4 (BANG10-SP)	dsc_{15}CsfD
CsuD	CS14 (WS3294A)	dsc_{19}CsuD
Class 5b antigens		
CsbD	CS17 (WS6788A)	dsc_{19}CsbD (reference)
CsbD AD	CS17 (WS6788A)	Adhesin domain (residues 19-205) of CsbD
CsbD E20738A	CS17 (E20738A)	CsbD has allelic variations of N62S/S74T/T84N/L85R/H144A/Y145N/Y293H in the CS17 allelic strain E20738A
CsbD LSN139	CS17 (LSN02-013966/A)	CsbD has allelic variations of L85I/H144A in the CS17 allelic strain LSN139
CsbD/T84N/L85R	CS17 (WS6788A)	CsbD mutant T84N/L85R
CsbD/H144A/Y145N	CS17 (WS6788A)	CsbD mutant H144A/Y145N

CsbD WS4240A	CS17 (WS4240A)	CsbD has allelic variation of L85I in the CS17 allelic strain of WS4240A
CooD	CS1 (E24377A)	dsc_{15}CooD
Class 5c antigens		
CotD	CS2 (C91f)	dsc_{19}CotD (reference)
CotD AD	CS2 (C91f)	Adhesin domain (residues 19-205) of CotD
CotD/R69A	CS2 (C91f)	CotD mutant R69A
CotD/T87A	CS2 (C91f)	CotD mutant T87A
CotD/K183A	CS2 (C91f)	CotD mutant K183A
CotD/R184A	CS2 (C91f)	CotD mutant R184A

Supplemental Figures

Figure S1. Anti-CfaE Mab ELISA to evaluate responses to various class 5 ETEC fimbrial adhesin variants. The responses to the immunogen CfaE were highlighted in black bars. The bars and error bars represent the respective mean OD values and standard deviations of at least two repeated assays. The dashed lines represented the limit of detection in the anti-CfaE Mab ELISA assays, which equals the sum of average background of PBS buffer and three times of the standard deviation. The mutated residue was recognized as a hotspot residue within the epitope when the mean OD value of the CfaE mutant was lower than the limit of detection.

A. anti-CfaE Mab P8D10 ELISA

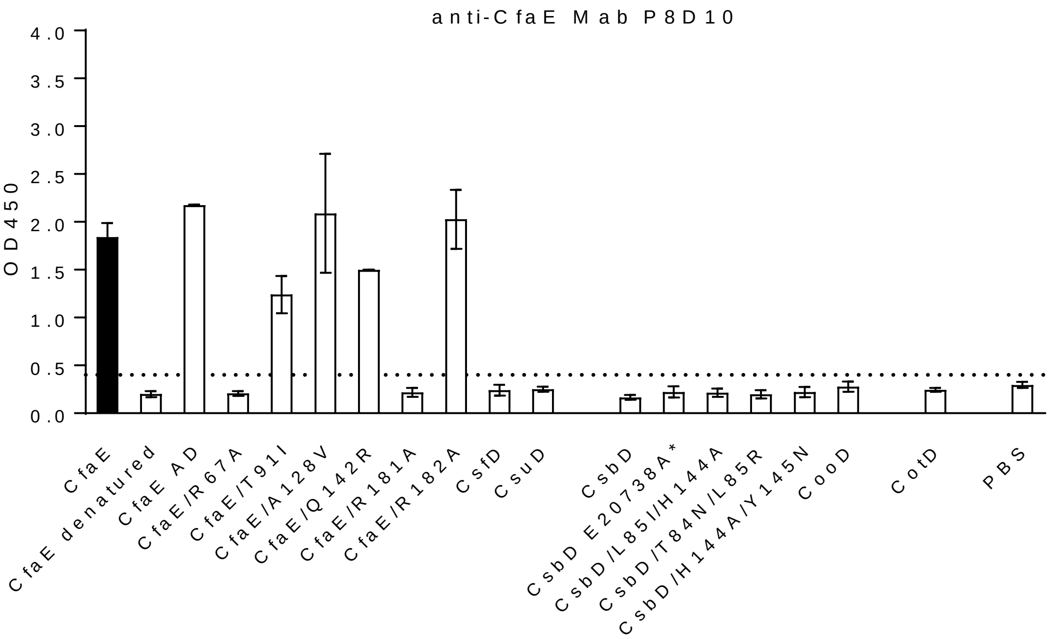

* CsbD E20738A has allelic variations of N62S/S74T/T84N/L85R/H144A/Y145N/Y293H comparing to CsbD in the reference strain WS6788A.

B. Anti-CfaE P6C11 ELISA

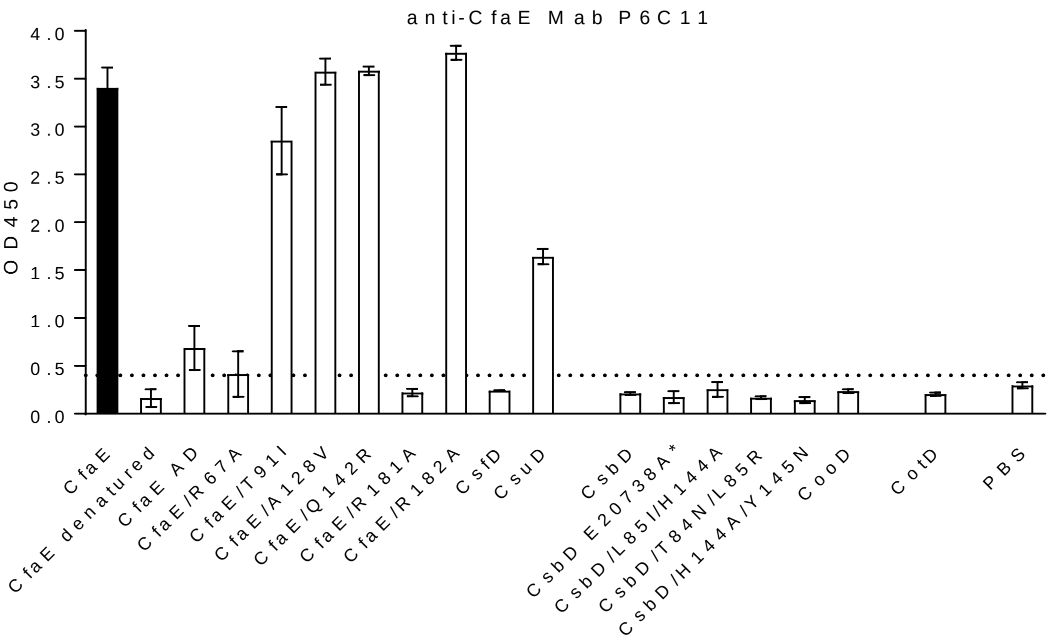

C. Anti-CfaE Mab P6H4 ELISA

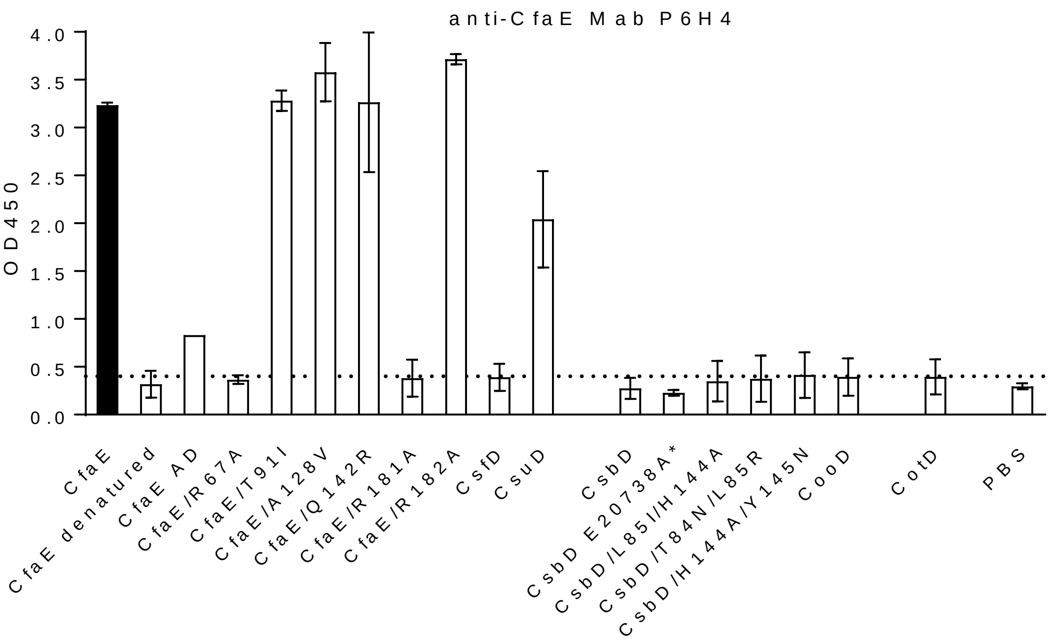

D. Anti-CfaE Mab P10A7 ELISA

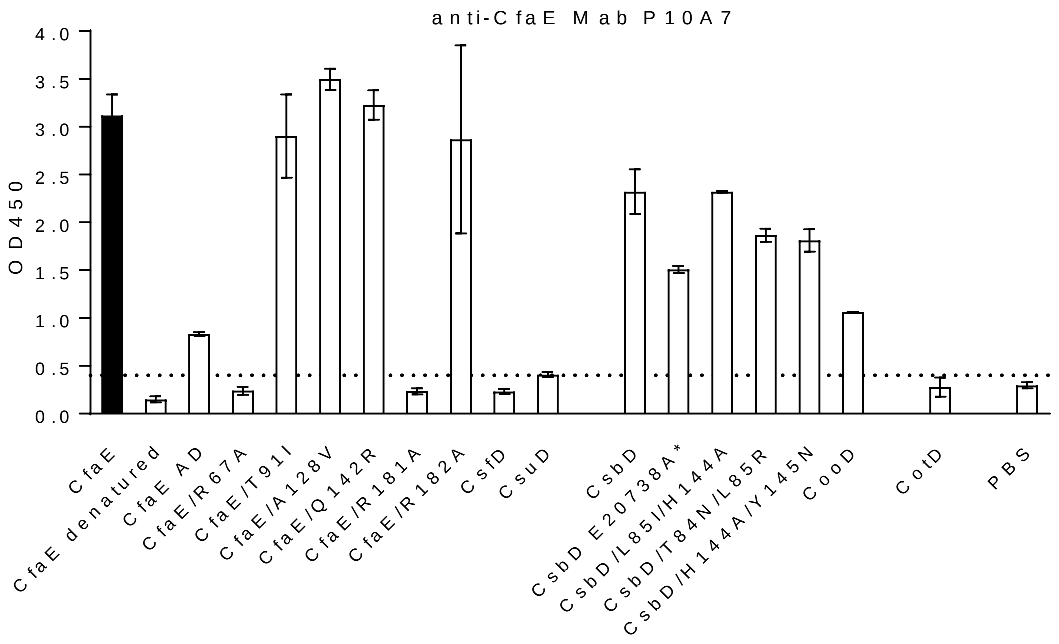

E. Anti-CfaE Mab P5C7 ELISA

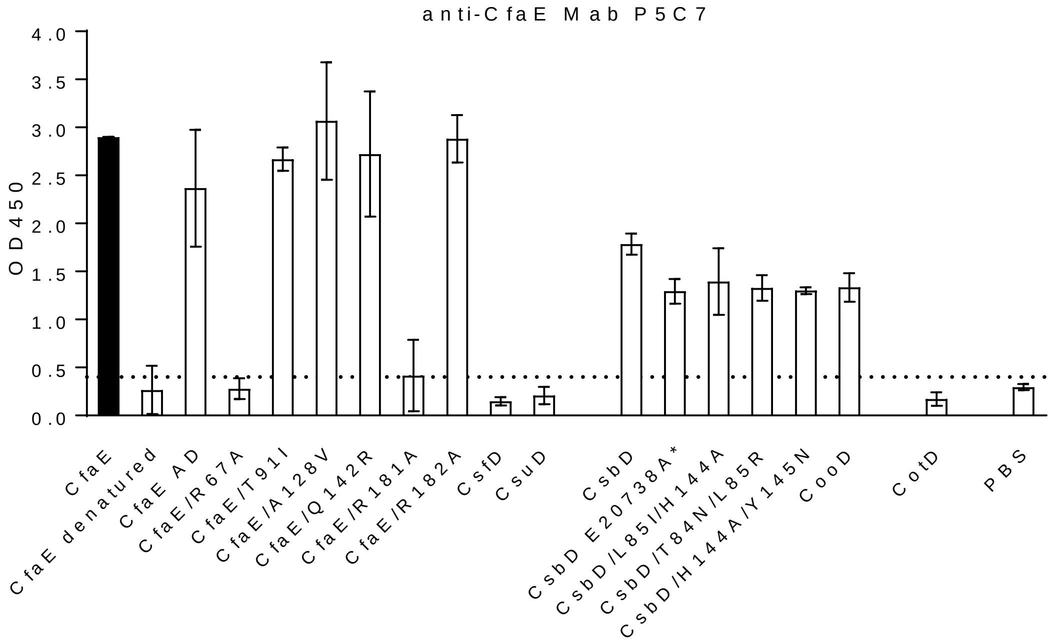

F. Anti-CfaE Mab P2E11 ELISA

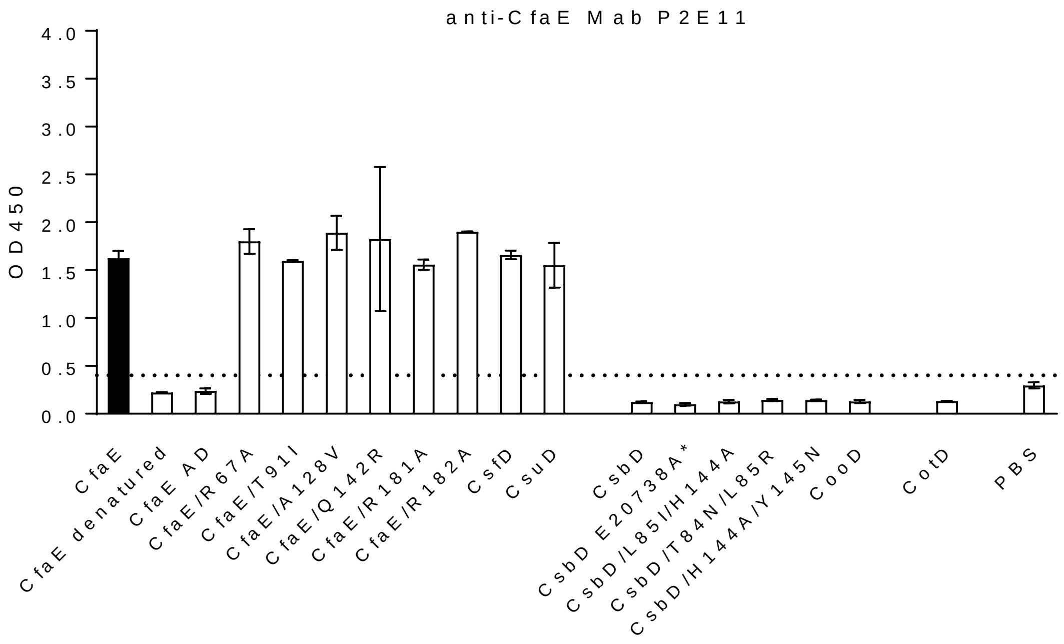

G. Anti-CfaE Mab P3B2 ELISA

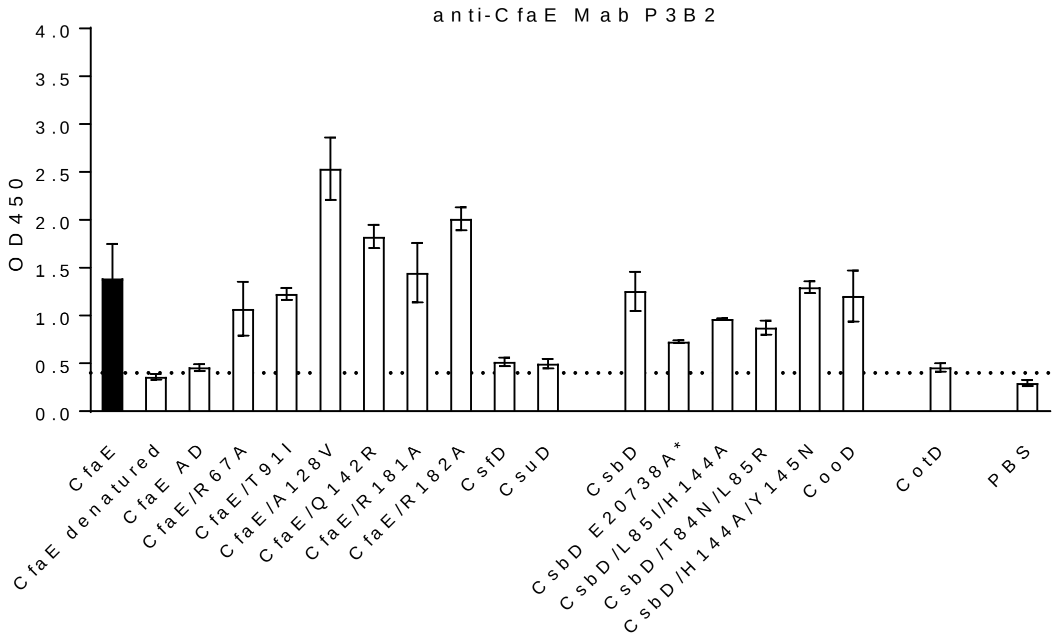

H. Anti-CfaE Mab P13A7 ELISA

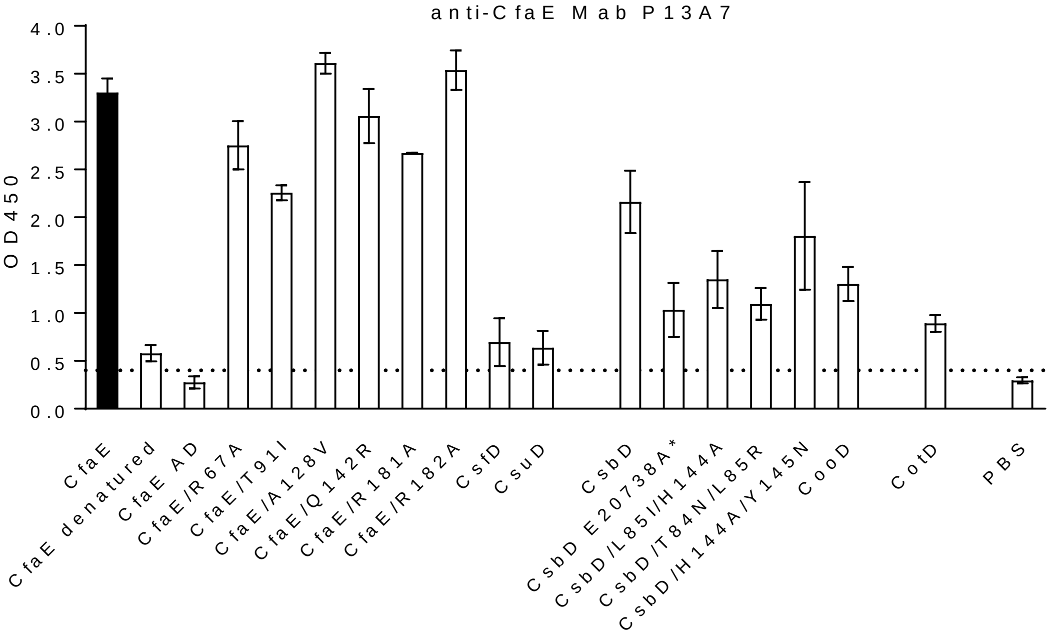

I. Anti-CfaE Mab P1F9 ELISA

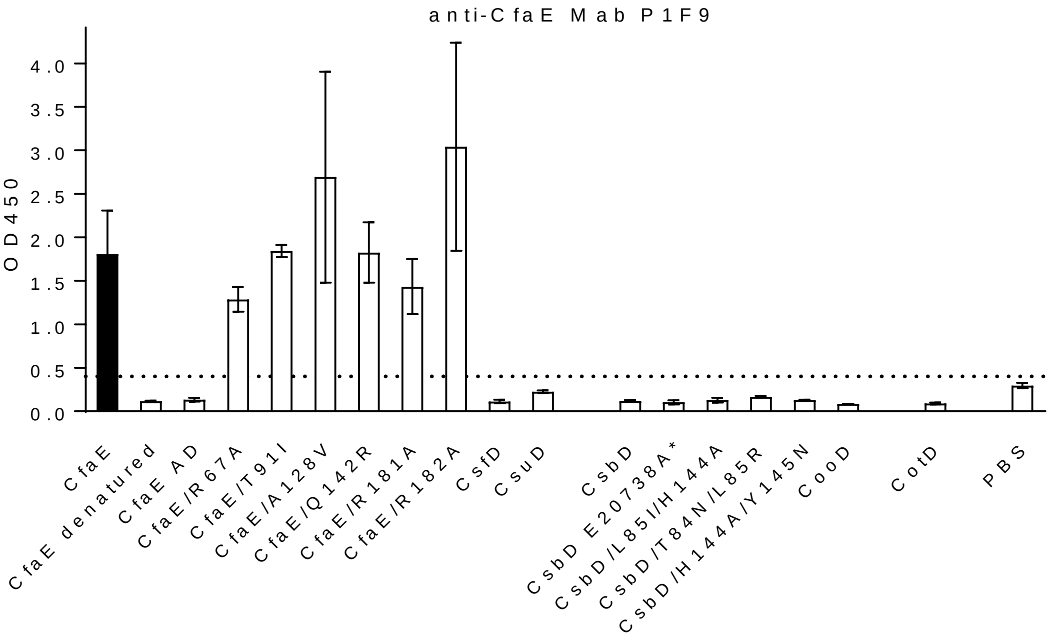

Figure S2. Anti-CsbD Mab ELISA to evaluate responses to various class 5 ETEC fimbrial adhesin variants. The responses to the immunogen CsbD were highlighted in black bars. The bars and error bars represent the respective mean OD values and standard deviations of at least two repeated assays. The dashed lines represented the limit of detection in the anti-CsbD Mab ELISA assays, which equals the sum of average background of PBS buffer and three times of the standard deviation. The mutated residue was recognized as a hotspot residue within the epitope when the mean OD value of the CsbD mutant was lower than the limit of detection.

A. Anti-CsbD Mab P7C2 ELISA

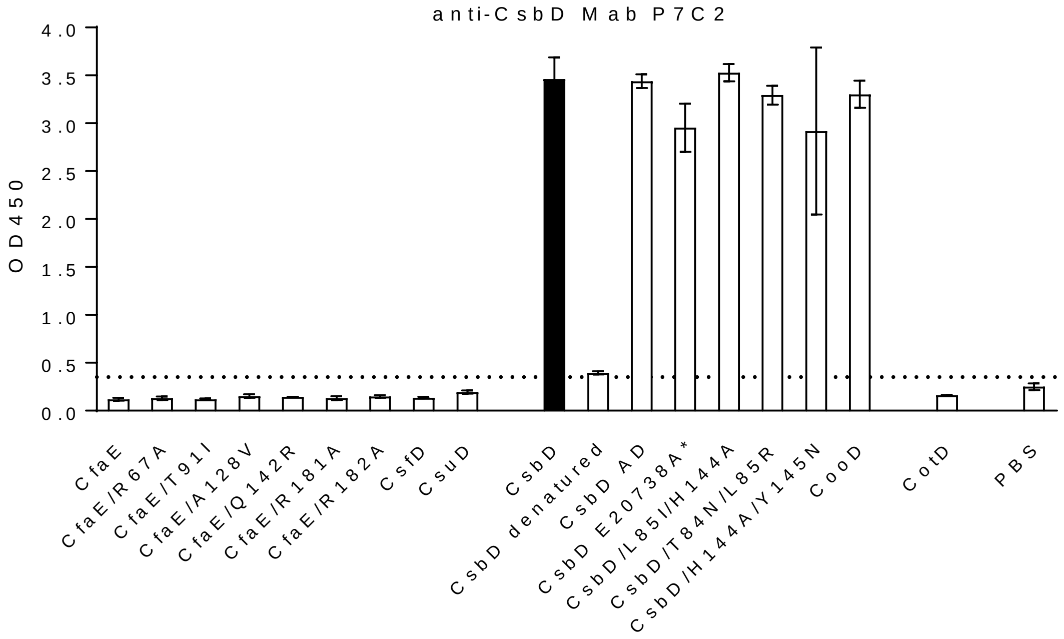

* CsbD E20738A has allelic variations of N62S/S74T/T84N/L85R/H144A/Y145N/Y293H comparing to CsbD in the reference strain WS6788A.

B. Anti-CsbD Mab P9A5 ELISA

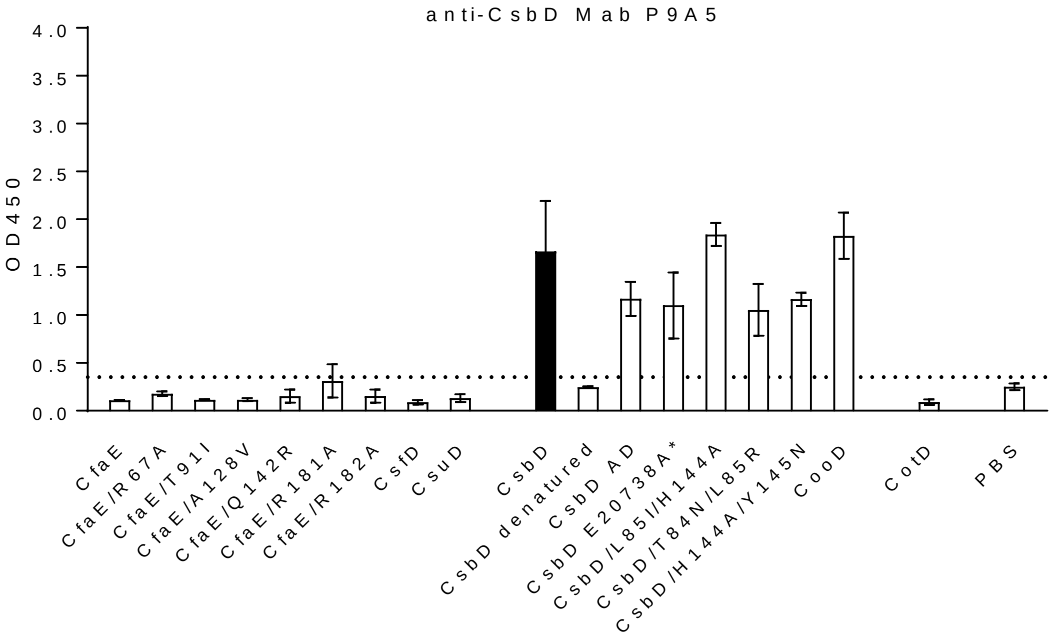

C. Anti-CsbD Mab P2H6 ELISA

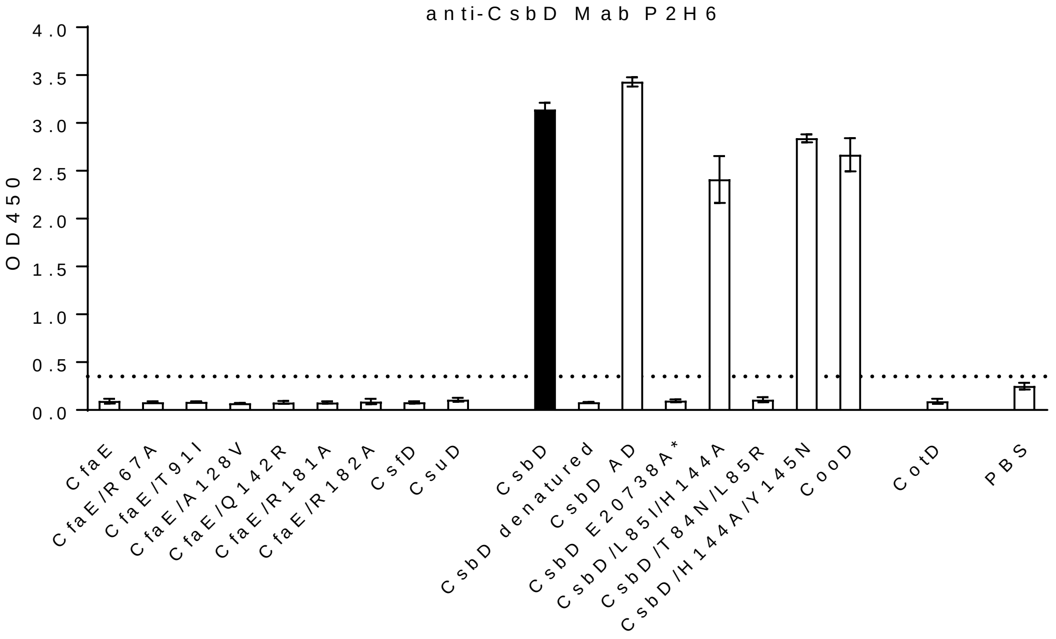

D. Anti-CsbD Mab P6G1 ELISA

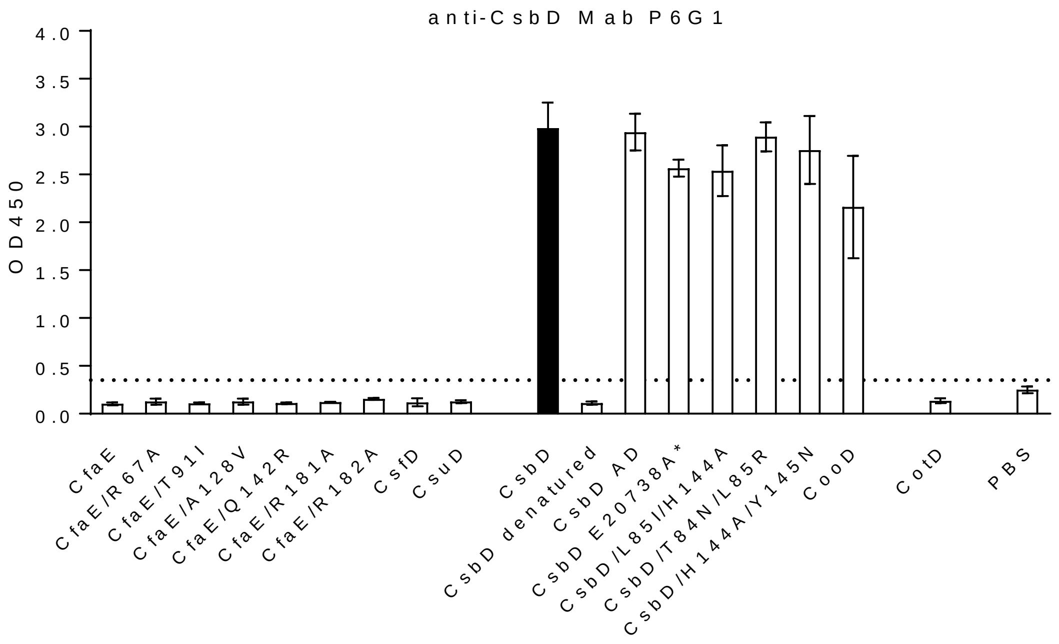

E. Anti-CsbD Mab P2A9 ELISA

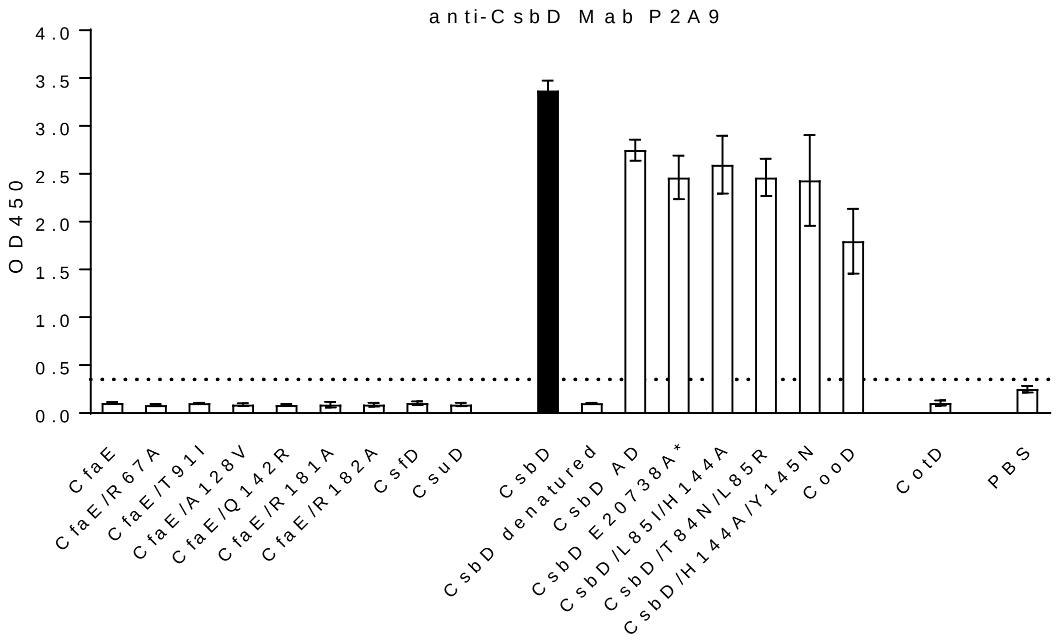

F. Anti-CsbD Mab P1F7 ELISA

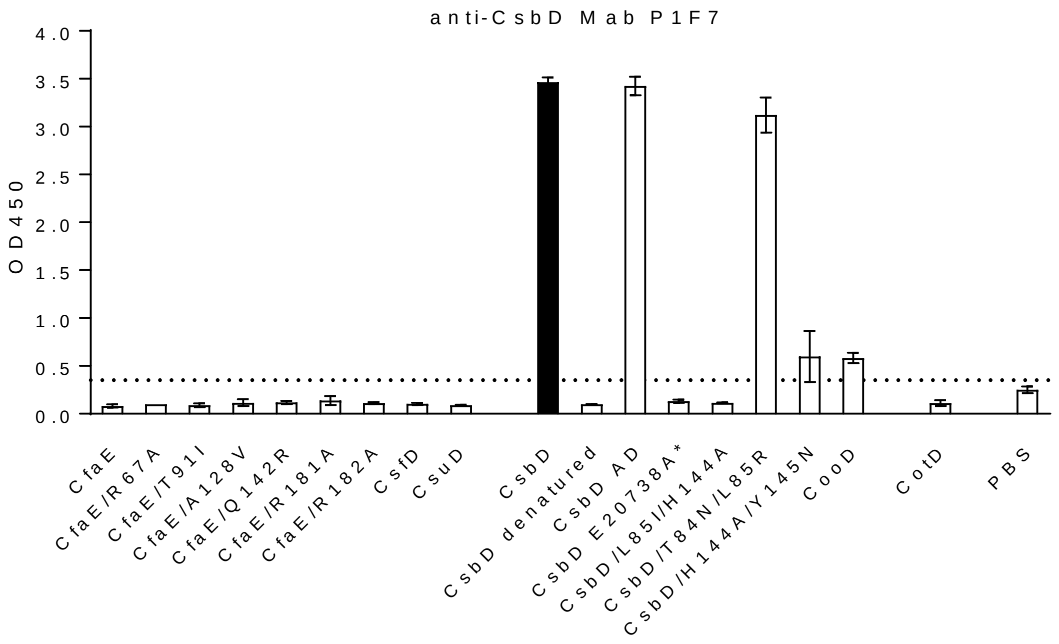

G. Anti-CsbD Mab P9E11 ELISA

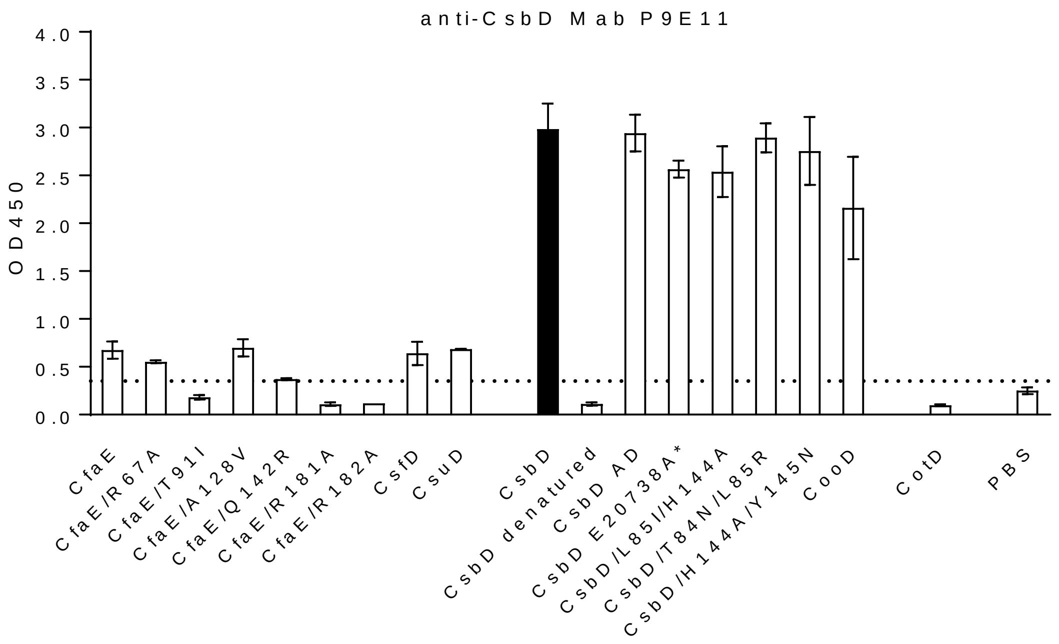

H. Anti-CsbD Mab P7F9 ELISA

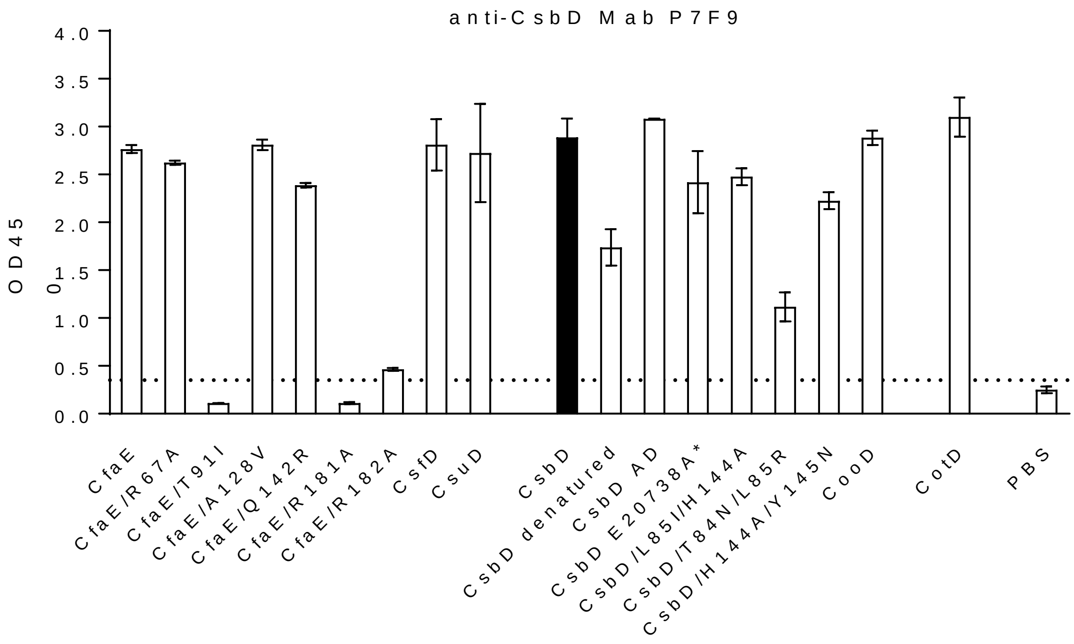

I. Anti-CsbD Mab P5A12 ELISA

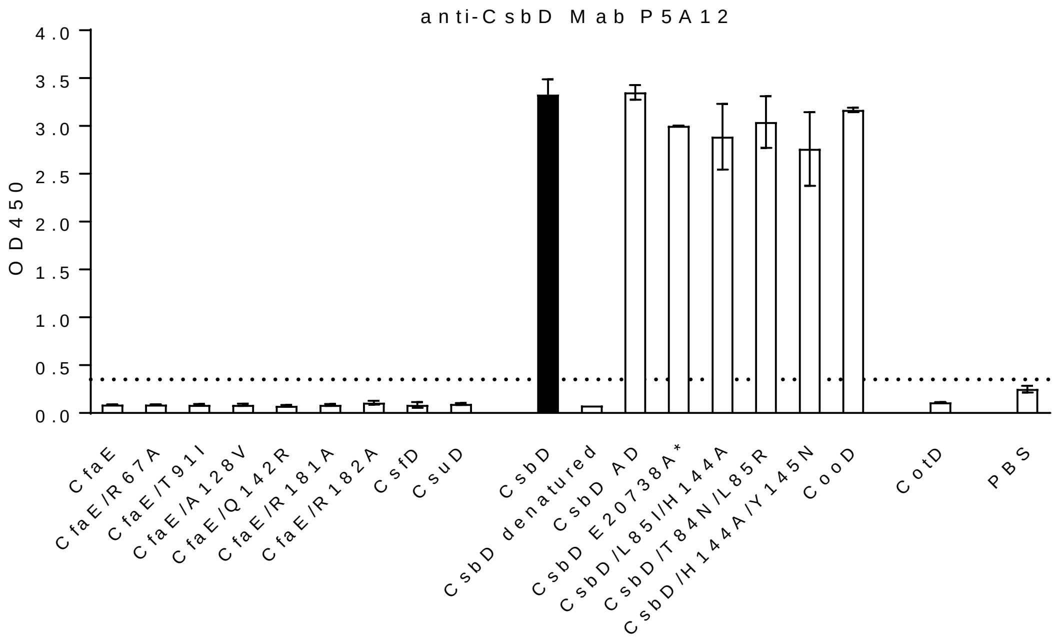

J. Anti-CsbD Mab P9D12 ELISA

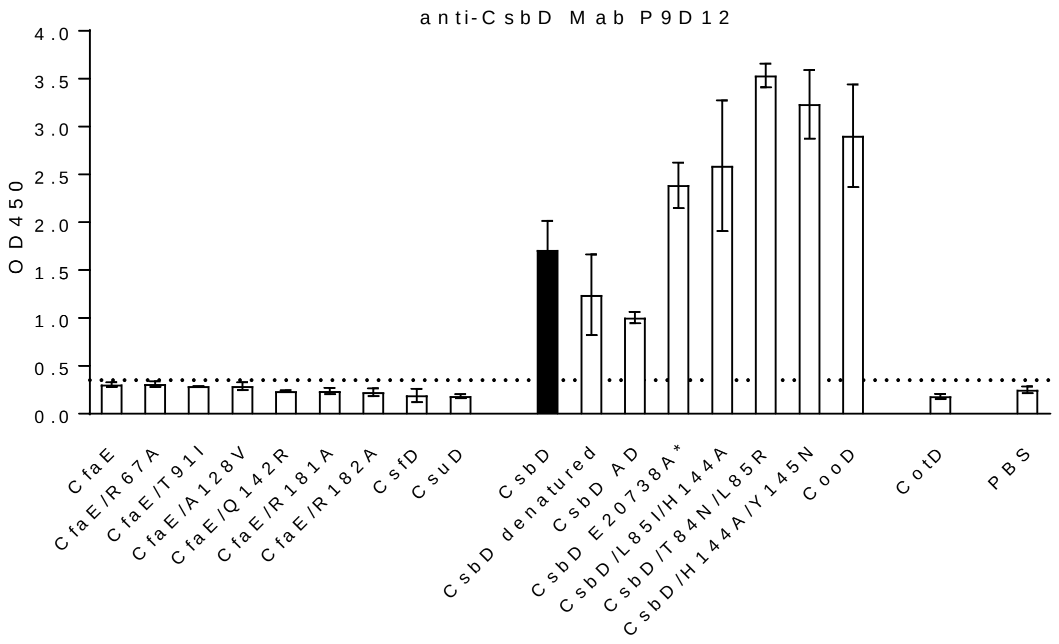

K. Anti-CsbD Mab P7F12 ELISA

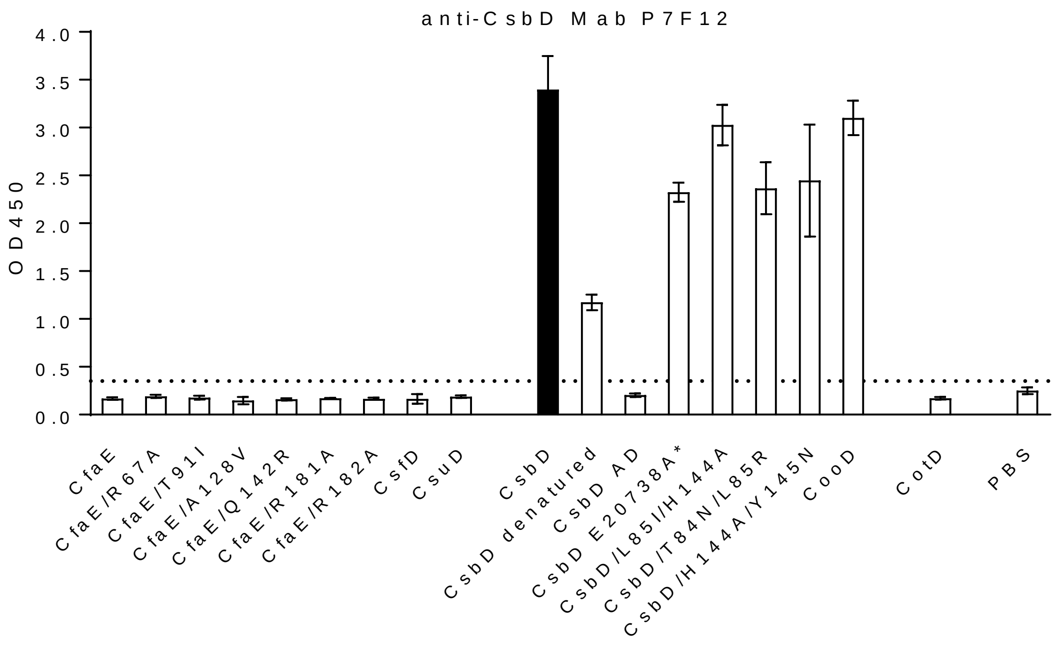

Figure S3. Anti-CotD Mab ELISA to evaluate responses to various class 5 ETEC fimbrial adhesin variants. The responses to the immunogen CotD were highlighted in black bars. The bars and error bars represent the respective mean OD values and standard deviations of at least two repeated assays. The dashed lines represented the limit of detection in the anti-CotD Mab ELISA assays, which equals the sum of average background of PBS buffer and three times of the standard deviation. The mutated residue was recognized as a hotspot residue within the epitope when the mean OD value of the CotD mutant was lower than the limit of detection.

A. Anti-CotD Mab P7F6 ELISA

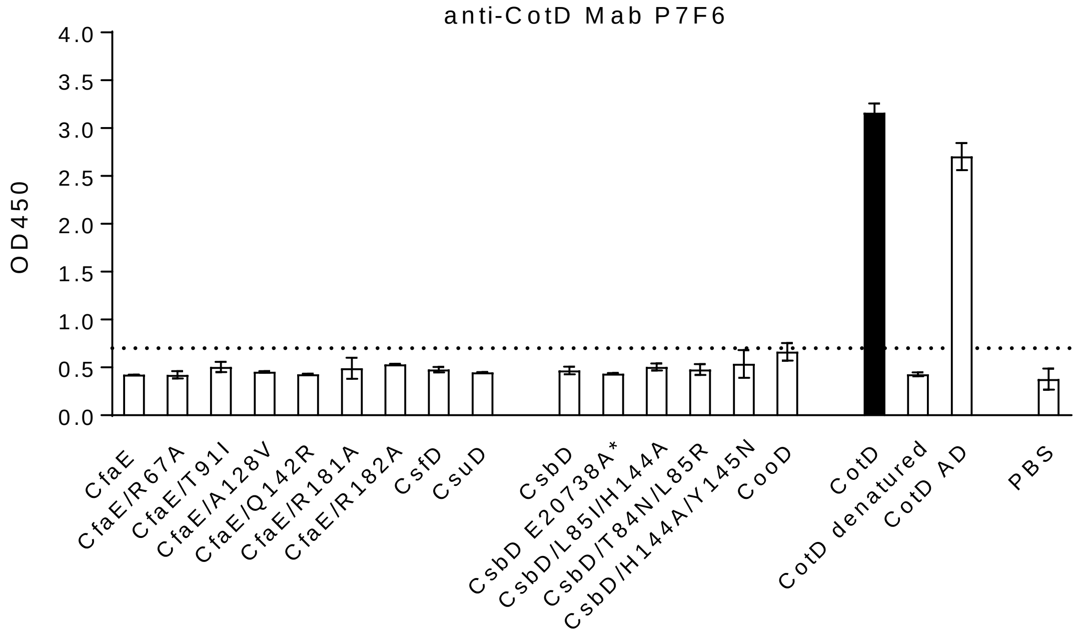

* CsbD E20738A has allelic variations of N62S/S74T/T84N/L85R/H144A/Y145N/Y293H comparing to CsbD in the reference strain WS6788A.

B. Anti-CotD Mab P3F4 ELISA

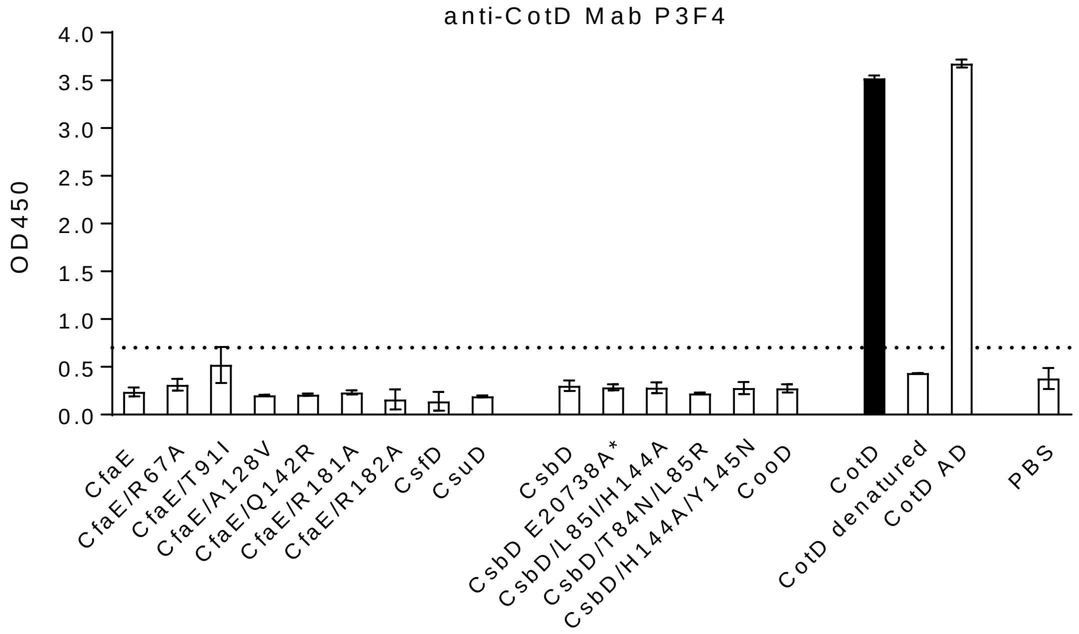

C. Anti-CotD Mab P6B8 ELISA

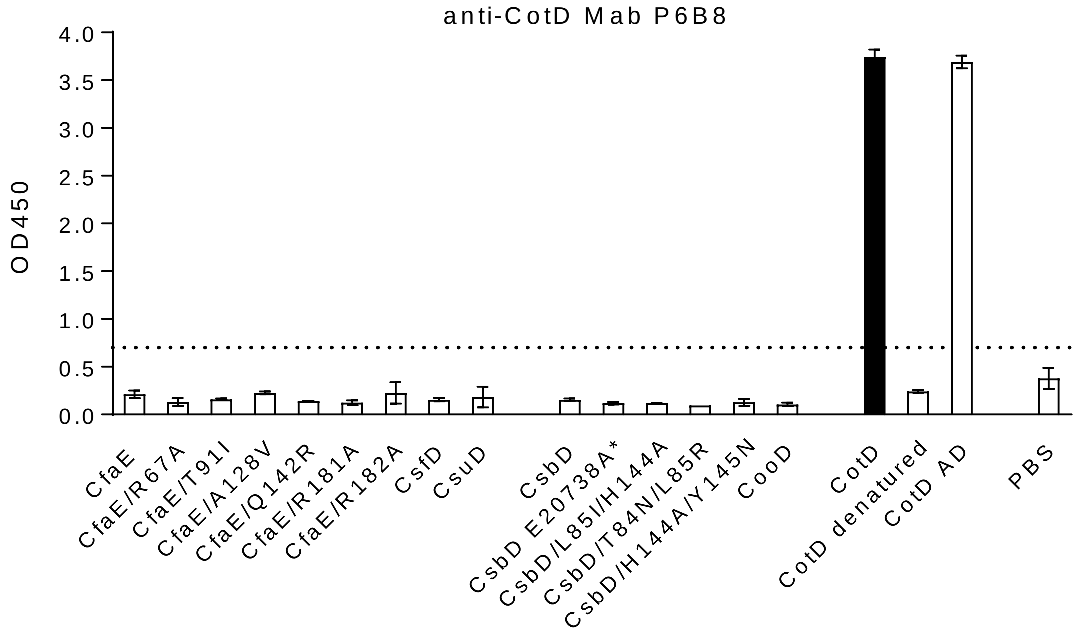

D. Anti-CotD Mab P3D11 ELISA

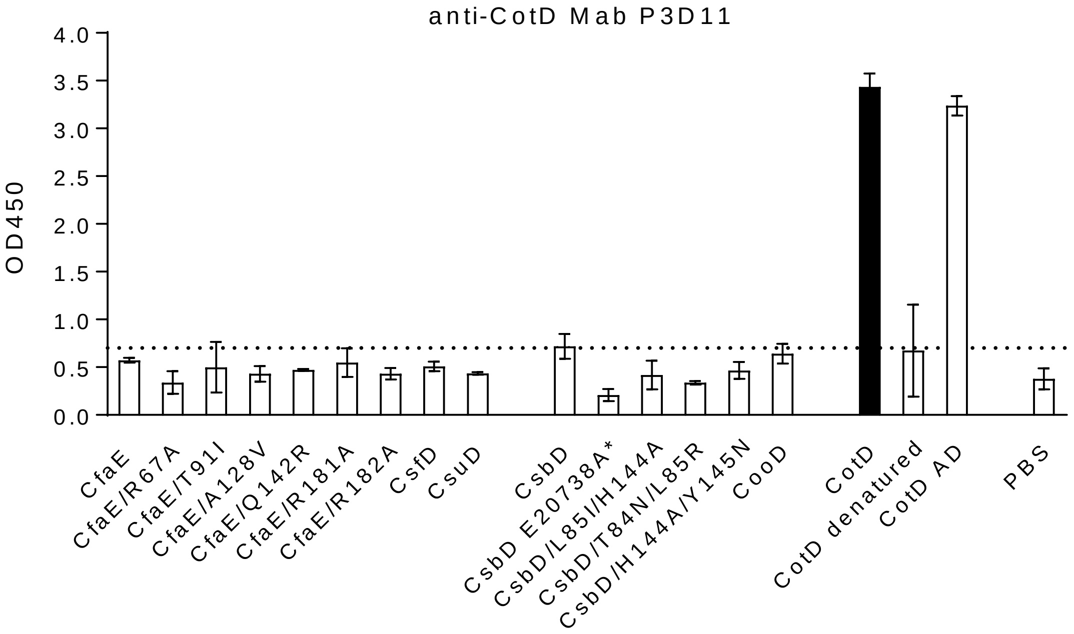

E. Anti-CotD Mab P9A10 ELISA

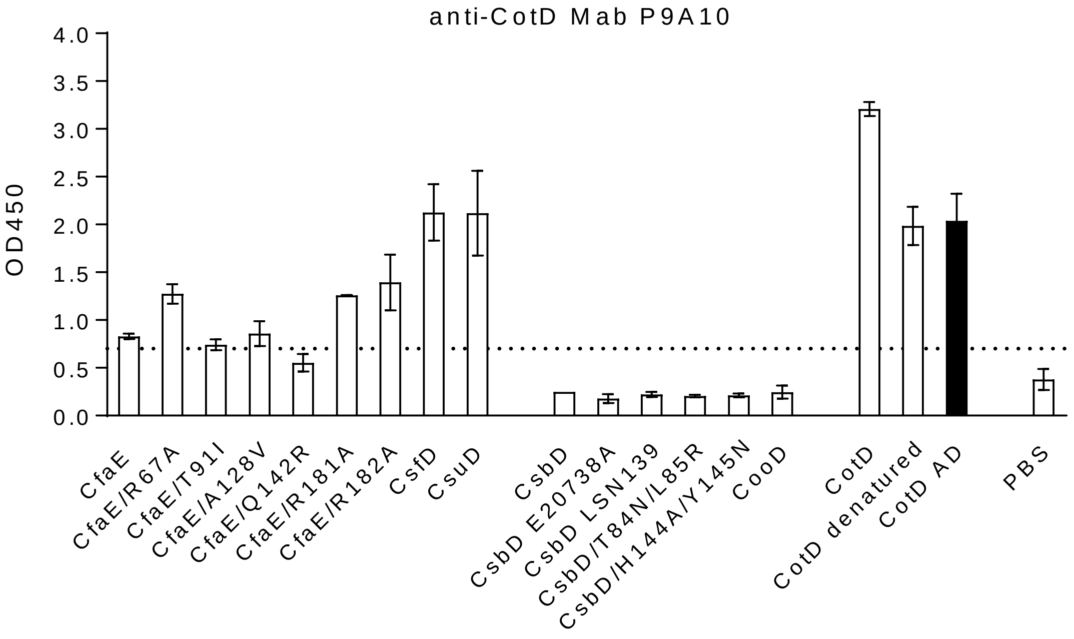

F. Anti-CotD Mab P9G7 ELISA

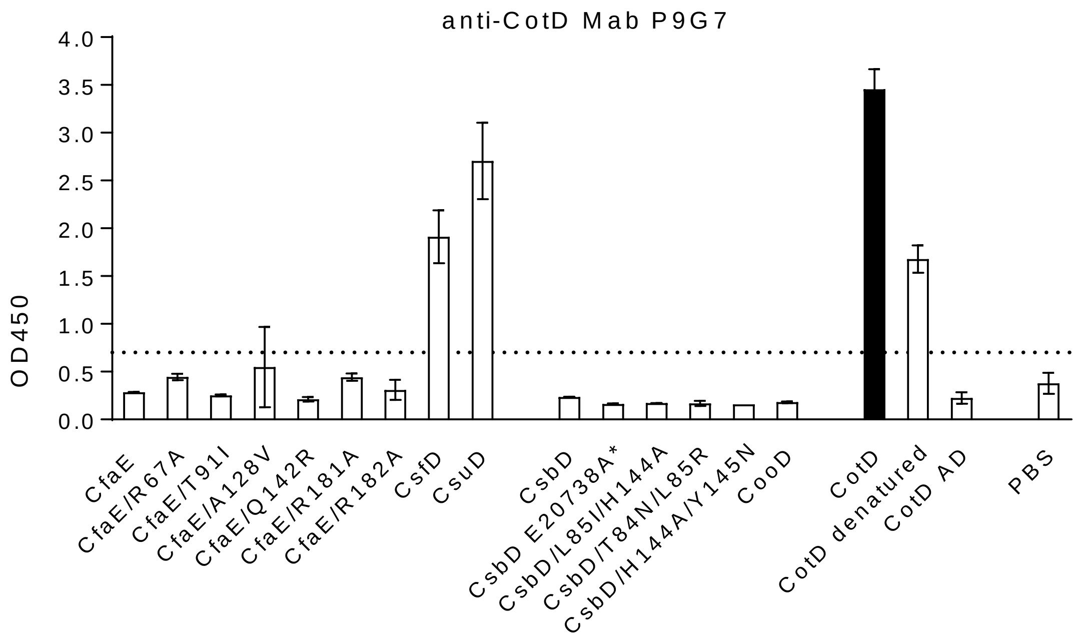

G. Anti-CotD Mab P2B8 ELISA

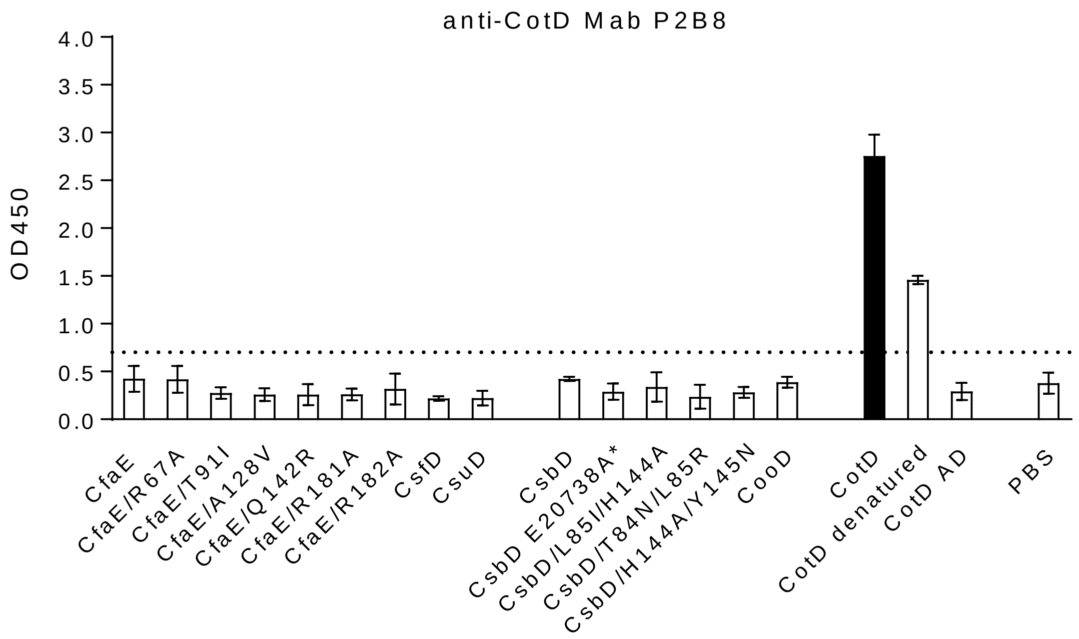

H. Anti-CotD mAb P12A2 ELISA

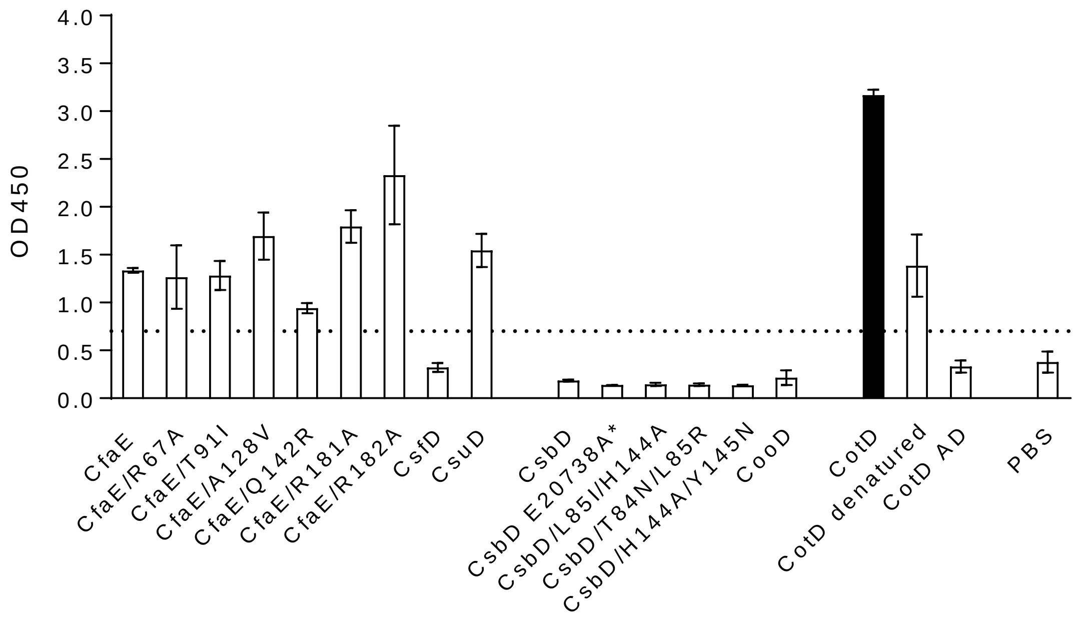

Figure S4. Additional anti-CotD mAb ELISA to evaluate responses to specific CotD adhesin mutants. The responses to the immunogen CotD were highlighted in black bars. The bars and error bars represent the respective mean OD values and standard deviations of at least three repeated assays. The dashed lines represented the limit of detection in the anti-CotD Mab ELISA assays, which equals the sum of average background of PBS buffer and three times of the standard deviation. The mutated residue was recognized as a hotspot residue within the epitope when the mean OD value of the CotD mutant was lower than the limit of detection.

A. Anti-CotD mAb P7F6 ELISA

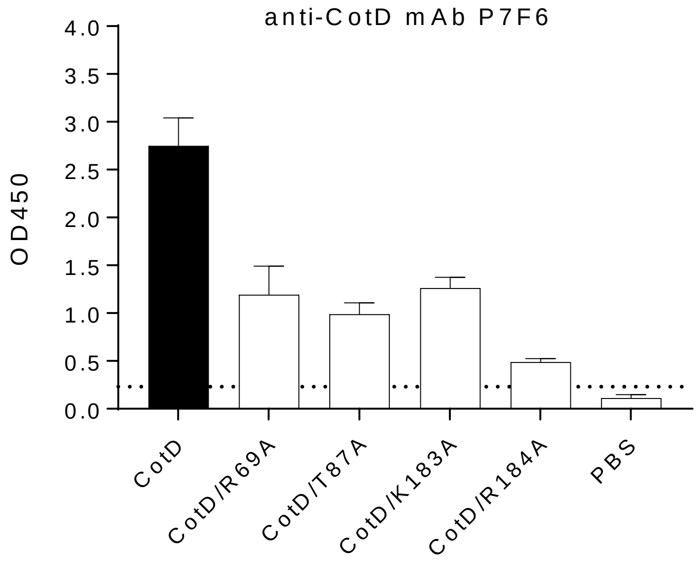

B. Anti-CotD mAb P3F4 ELISA

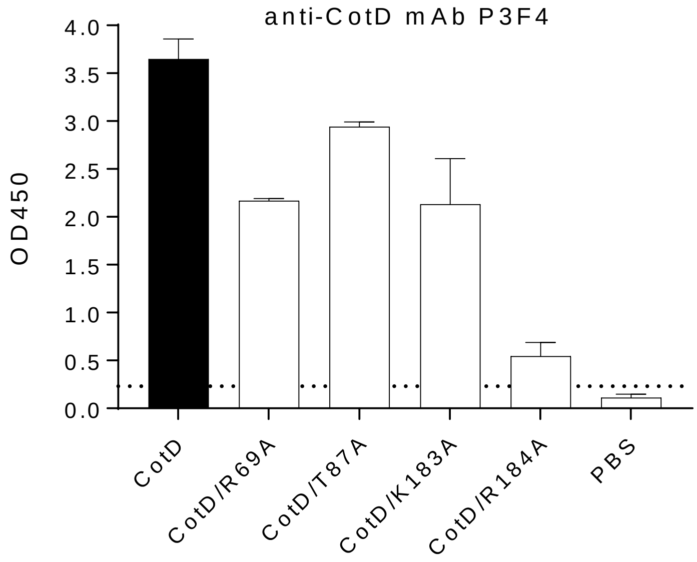

C. Anti-CotD mAb P6B8 ELISA

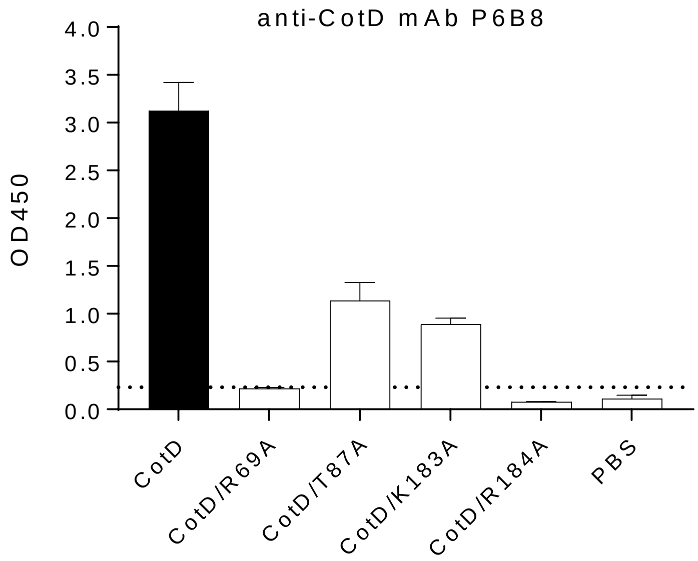

D. Anti-CotD mAb P3D11 ELISA

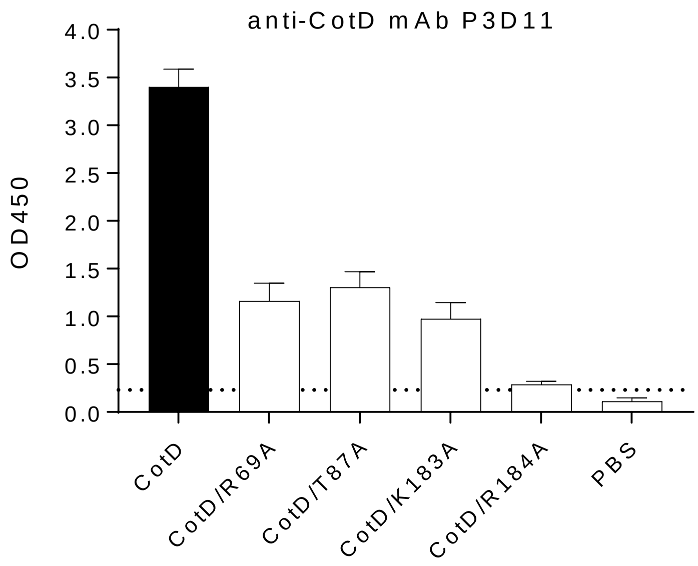

E. Anti-CotD mAb P9A10 ELISA

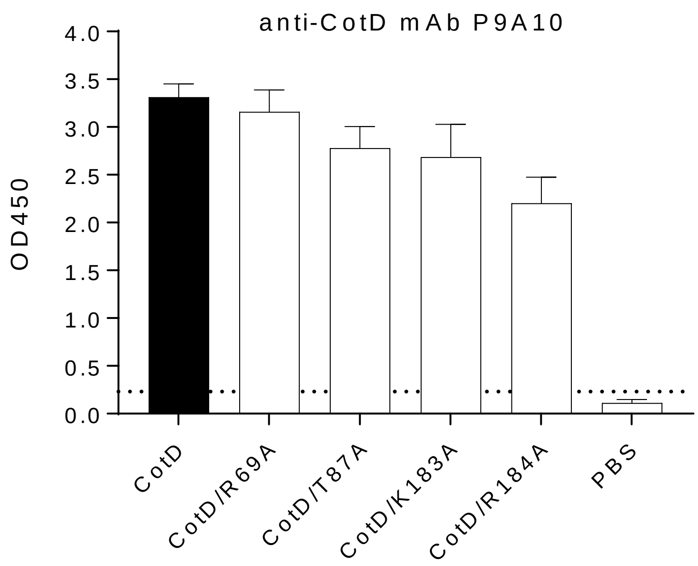

F. Anti-CotD mAb P9G7 ELISA

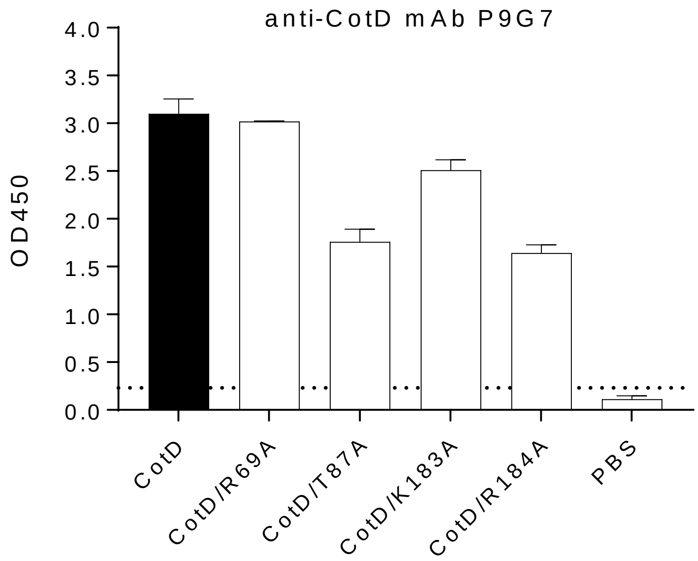

G. Anti-CotD mAb P2B8 ELISA

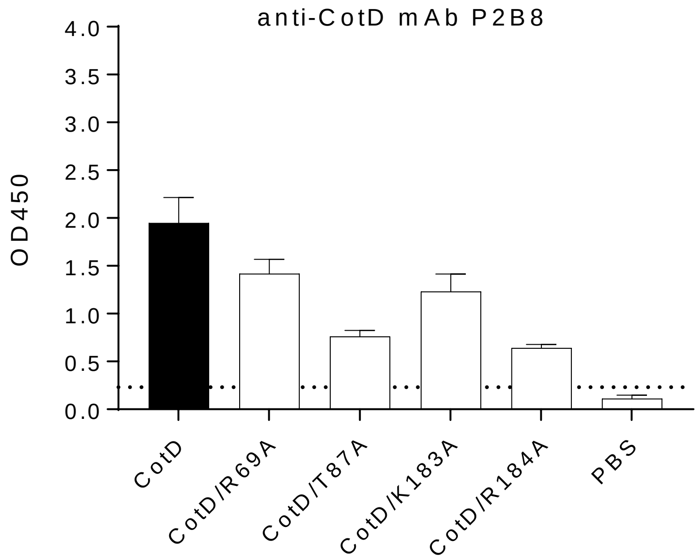

H. Anti-CotD mAb P12A2 ELISA

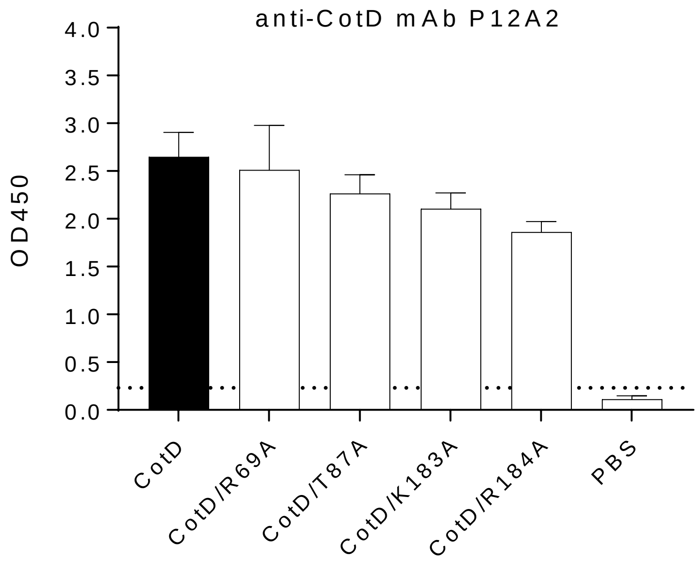

Figure S5. Multiple sequence alignment of CfaE from CFA/I H10407 strain, CsbD from CS17 WS6788A strain, and CotD from CS2 C91f strain. Residues with allelic variations and site-directed mutations are highlighted with black boxes. The numbers at the top of the sequences indicate amino acid positions in CfaE starting from the leader sequence (not shown). The numbers on the right side of the sequences indicate amino acids positions starting from the leader sequences (not shown) of CfaE, CsbD, and CotD, respectively.

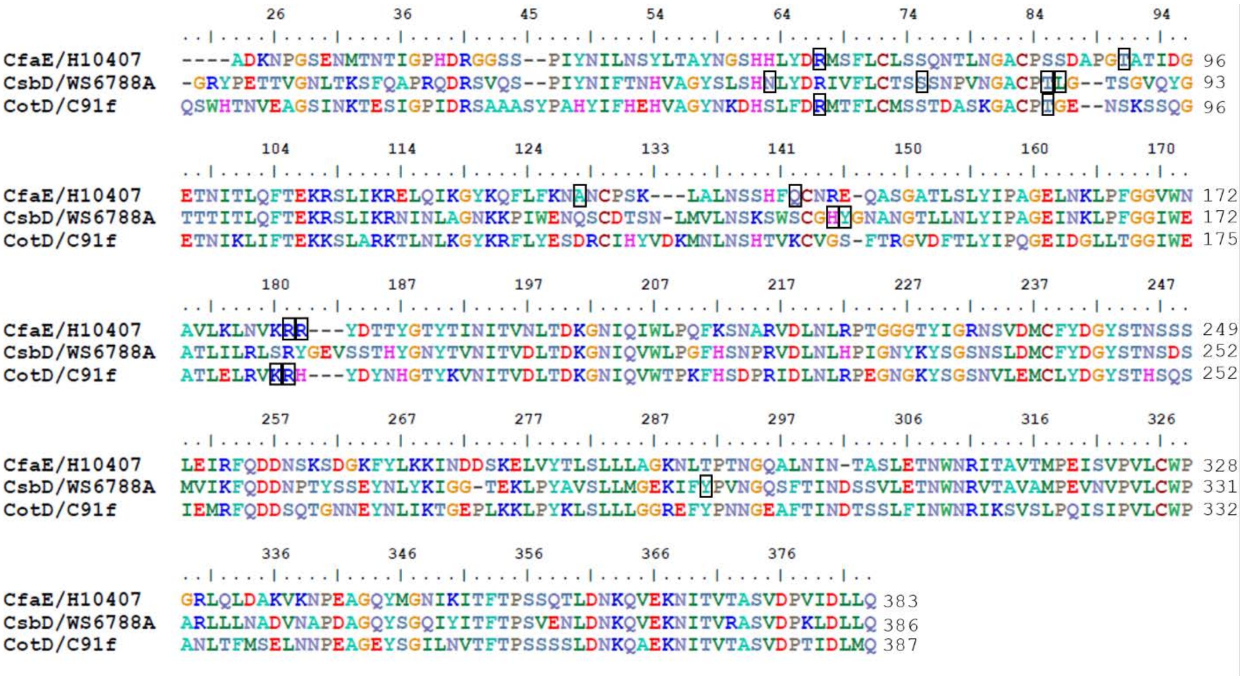

Figure S6. Anti-CfaE Mab ELISA to evaluate responses to two domains of CfaE. The responses to the immunogen CfaE were highlighted in black bars. The bars and error bars represent the respective mean OD values and standard deviations of at least two repeated assays. The dashed lines represented the limit of detection in the ELISA assay, which equals the sum of average background of PBS buffer and three times of the standard deviation.

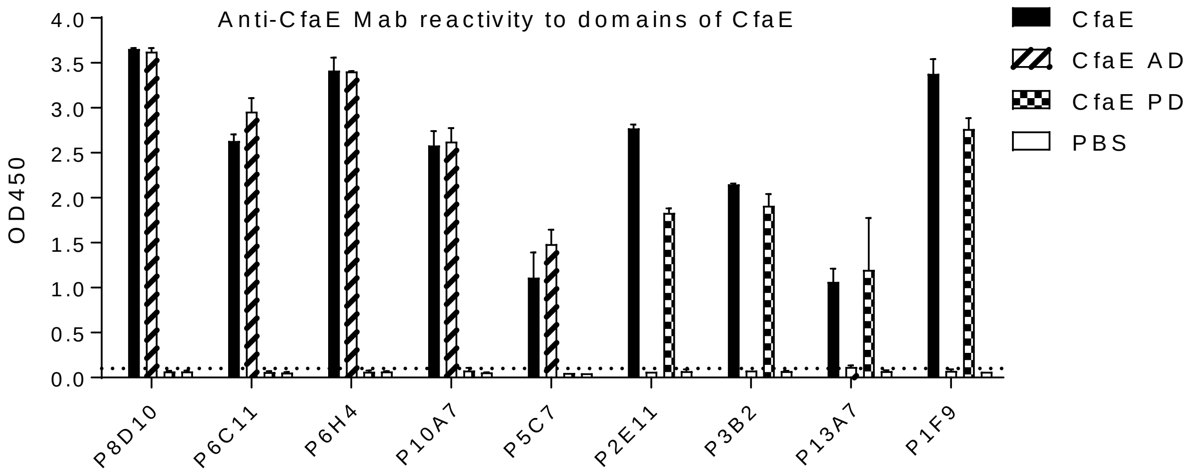

Supplement: Supplemental file 1 [file IAI.00246-20-s0001.pdf]
